# Cost-effectiveness of first-line immunotherapy combinations with or without chemotherapy for advanced non–small cell lung cancer: a modelling approach

**DOI:** 10.1186/s12885-023-10938-8

**Published:** 2023-05-15

**Authors:** Wen Hui, Ruomeng Song, Hongyu Tao, Zhixiang Gao, Min Zhu, Mingyue Zhang, Huazhang Wu, Daichen Gong, Xiyan Zhang, Yuanyi Cai

**Affiliations:** 1 West China Hospital, Sichuan University, Chengdu, China; 2 School of Public Health, China Medical University, Shenyang, China; 3 Laboratory of Oncology, Institute of Medicinal Biotechnology, Chinese Academy of Medical Sciences and Peking Union Medical College, Beijing, China; 4 Department of Pharmacy, Affiliated Central Hospital of Shenyang Medical College, Shenyang, China; 5 Department of Health Service Management, School of Health Management, China Medical University, Shenyang, China; 6 Department of Health Economics, School of Health Management, China Medical University, Shenyang, China; 7 Medical Record Department, Xiamen Humanity Hospital, Xiamen, China

**Keywords:** Cost-effectiveness, Non-small cell lung cancer, Immunotherapy combination, Hazard ratio, Partitioned survival model

## Abstract

**Background:**

Many studies have explored the cost-effectiveness of immunotherapy versus chemotherapy alone. However, there is paucity of evidence on direct pharmacoeconomic studies related to immunotherapy combinations. Thus, we aimed at assessing the economic outcomes of first-line immunotherapy combinations in the treatment of advanced non-small cell lung cancer (NSCLC) from the Chinese health care perspective.

**Methods:**

The mutual hazard ratios (HRs) of ten immunotherapy combinations and one chemotherapy regimen for the overall survival (OS) and progression-free survival (PFS) were obtained from a network meta-analysis. Based on proportional hazard (PH) assumption, adjusted OS and PFS curves were established to make the effects comparable. With the parameters of cost and utility, and of scale and shape from the fit of adjusted OS and PFS curves obtained from previous studies, a partitioned survival model was designed to estimate the cost-effectiveness of immunotherapy combinations versus chemotherapy alone. Parameter uncertainty in model inputs was assessed using one-way deterministic and probabilistic sensitivity analyses.

**Results:**

The incremental cost of camrelizumab plus chemotherapy versus chemotherapy alone was $13,180.65, the lowest among all the other immunotherapy combinations. Furthermore, sintilimab plus chemotherapy (sint-chemo) provided the highest quality-adjusted life-year (QALY) benefit versus chemotherapy alone (incremental QALYs = 0.45). Sint-chemo yielded the best incremental cost-effectiveness ratio (ICER) versus chemotherapy alone (ICER = $34,912.09/QALY), at the current price. The cost-effectiveness probabilities were 32.01% and 93.91% for pembrolizumab plus chemotherapy, and atezolizumab plus bevacizumab plus chemotherapy, respectively (if the original price of the pembrolizumab, atezolizumab, and bevacizumab were decreased by 90%).

**Conclusions:**

Based on the fact that there is fierce competition in the PD-1/PD-L1 market, pharmaceutical enterprises should strive for greater efficacy, and optimal pricing strategy for therapies.

**Supplementary Information:**

The online version contains supplementary material available at 10.1186/s12885-023-10938-8.

## Background

Lung cancer is a major global health issue [1]. According to the GLOBOCAN 2020 database, the incidence of lung cancer was 2.207 million, accounting for 11.4% of all cancer cases. The number of deaths owing to lung cancer was 1.796 million, taking up 18.0% of all deaths. Compared with female breast cancer, lung cancer is the most widespread and is the leading cause of death due to cancer in males, with an estimated 1.436 million new cases and 1.189 million deaths in 2020 [2]. In the two broad histologic subtypes of lung cancer, non-small cell lung cancer (NSCLC) constitutes approximately 85–90% [3]. Furthermore, 60–70% of patients with lung cancer are often diagnosed at stages III and IV where an overall 5-year survival rate is dismal [4–6]. Therefore, improving survival and quality of life for patients with advanced NSCLC is of utmost relevance.

In the past decades, immunotherapies have led to prolong overall survival (OS) and progression-free survival (PFS). Recent studies showed that first-line immunotherapy combinations regimen, including immune checkpoint inhibitors (ICI) plus chemotherapy, ICI with anti-angiogenesis drugs, and ICI combinations achieve better clinical efficacy compared with that obtained with chemotherapy in KEYNOTE-189 trial, IMpower150 trial, and CheckMate 9LA trial [7–9]. However, in the phase 3 MYSTIC trial, immunotherapy combinations did not improve OS compared with that of chemotherapy alone [10]. Because of the uncertainty of results, a series of meta-analyses were conducted in recent years [11–15]. Among them, several studies were also performed to further demonstrate the clinical benefit of currently available first-line immunotherapy combination in the treatment of patients with advanced NSCLC [16–18]. They demonstrated that immunotherapy combinations lead to higher efficacy, and improve clinical practice to some extent.

However, only considering clinical efficacy and safety could not meet the demand of real decision-making practice. Economic burden are also the important factors for decision makers. Compared with chemotherapy, the economic burden of immunotherapy combination is dramatically increasing from the perspective of payers including national health care insurance payers, commercial payers, and patients. Although previous studies have evaluated the cost-effectiveness of several immunotherapy regimens [19, 20], an optimal cost-effective immune agent has not yet been elucidated owing to the limited number of immune drugs involved in each study. To facilitate health care decision, a comprehensive comparison of the cost-effectiveness of all immunotherapy combinations is necessary.

Thus, through this study, we aimed at exploring the cost-effectiveness of first-line immunotherapy combinations in the treatment of advanced NSCLC from the perspective of the Chinese health care system, in order to facilitate clinical practice and policy making, thereby creating a reference for developing countries.

## Methods

This cost-effectiveness analysis was conducted according to the Consolidated Health Economic Evaluation Reporting Standards 2022 (CHEERS 2022) statement, which was the guidance for health economic evaluation [21].

### Target population and clinical treatments

The target population was patients with advanced squamous or non-squamous NSCLC confirmed either histologically or cytologically, which was based on a published network meta-analysis involving 16 studies, 8278 patients, 10 immunotherapy combinations, and 1 chemotherapy alone. The immunotherapy regimens included sintilimab plus chemotherapy (sint-chemo), pembrolizumab plus chemotherapy (pem-chemo), nivolumab plus ipilimumab plus chemotherapy (nivo-ipi-chemo), tislelizumab plus chemotherapy (tisle-chemo), camrelizumab plus chemotherapy (camre-chemo), nivolumab plus ipilimumab (nivo-ipi), atezolizumab plus bevacizumab plus chemotherapy (atezo-beva-chemo), durvalumab plus tremelimumab plus chemotherapy (durva-treme-chemo), atezolizumab plus chemotherapy (atezo-chemo), and durvalumab plus tremelimumab (durva-treme) [22].

### Survival analysis

Through the network meta-analysis, we obtained the hazard ratios (HRs) of PFS and OS among the 11 treatment regimens (10 immunotherapy combinations and 1 chemotherapy alone). We selected atezo-chemo from the IMpower130 trial as the baseline treatment due to its large sample, long follow-up time, and stable result [23]. The survival function relating atezo-chemo and 10 other treatment regimens was based on the following derivation (A or B represented for any 1 of the 11 treatment regimens.):

1 $$h{\left(t\right)}_{A}=HR*h{\left(t\right)}_{B}$$

2 $${\int }_{0}^{t}h{\left(u\right)}_{A}du = {\int }_{0}^{t}HR *h{\left(u\right)}_{B}du=HR{\int }_{0}^{t}h{\left(u\right)}_{B}du$$

3 $${H\left(t\right)}_{A} =HR * {H\left(t\right)}_{B}$$

4 $${e}^{{-H\left(t\right)}_{A}} = {e}^{-{HR*H\left(t\right)}_{B}} = {\left[{e}^{{-H\left(t\right)}_{B}}\right]}^{HR}$$

5 $${s\left(t\right)}_{A} = {\left[{s\left(t\right)}_{B}\right]}^{HR}$$

We used WebPlotDigitizer to obtain the data of PFS and OS curves in the intervention arm of atezo-chemo in the IMpower130 trial. The individual patient data (IPD) of PFS and OS for the other nine immunotherapy combinations and one chemotherapy regimen was determined, based on Eq. (5), with the assumption of the same initially enrolled patients, and number lost to follow-up, thereby controlling the baseline characteristics, and making the treatment regimen become the only difference among the 11 treatment regimens.

Then the IPD of atezo-chemo arm were input into R (V4.0.3), and matched with the best distribution through survHE package. According to the Akaike information criterion (AIC), Bayesian information criterion (BIC), and visual inspection, log-logistic distribution which was an accelerated failure time (AFT) model was selected for both OS and PFS curve fit in the atezo-chemo arm (Table S1 and Figure S1-S4) [24]. Based on the proportional hazard (PH) assumption, the log-logistic distribution was also used to fit and extrapolate the PFS and OS curves for the other ten treatment regimens. The scale and shape parameters of all the eleven log-logistic distributions for PFS or OS curve were set as follows (x = 0 represented for the control arm, x = 1 represented for the intervention arm.):

6 $$s\left(t|x\right) = {s}_{0}\left({t*e}^{-\beta x}\right)$$

In the package of “flexsurv” in R language

7 $$s\left(t\right) = \frac{1}{\left[1+{\left(\frac{t}{scale}\right)}^{shape}\right]}$$

8 $$s\left(t|x=0\right) = {s}_{0}\left(t\right) = \frac{1}{\left[{1+\left(\frac{t}{scale}\right)}^{shape}\right]}$$

9 $$s\left(t|x=1\right) = {s}_{0}\left({t*e}^{-\beta }\right) = \frac{1}{\left[{1+\left(\frac{t}{{e}^{\beta }*scale}\right)}^{shape}\right]}$$

10 $${scale}_{arm=1} = {scale}_{arm=0} * {e}^{\beta }$$

On the basis of the derivation above, the common shape of the OS curve and the common shape of the PFS curve for the 11 treatment regimens were 1.3 and 1.7 respectively. The scales of OS and PFS curves for the 11 treatment regimens were presented in Table 1.

**Table 1:** Scale of OS and PFS curves in the immunotherapy combination group and chemotherapy group

Intervention	OS curve	PFS curve
Chemo	14.6	5.1
Sint-chemo	24.2	8.9
Pem-chemo	23.5	8.5
Nivo-ipi-chemo	21.8	7.0
Tisle-chemo	20.8	8.5
Camre-chemo	19.9	7.8
Nivo-ipi	19.7	6.1
Atezo-beva-chemo	20.0	10.3
Durva-treme-chemo	17.6	5.8
Atezo-chemo	17.4	7.2
Durva-treme	15.4	4.3

### Model overview

**Fig. 1:**
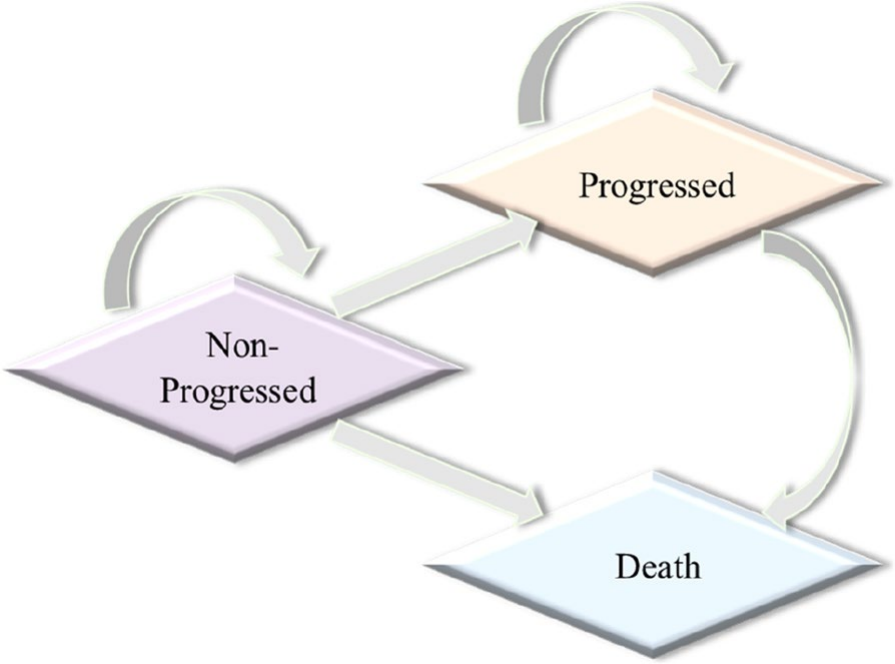
Three main health states assumption of patients with NSCLC in the model

We constructed a partitioned survival model, which included three states (progression-free survival, progressive disease, and death) to portray disease progression (Fig. 1). Each cycle length was set at 3 weeks, and the time horizon was 10 years, considering the poor prognosis for advanced NSCLC. The main outcomes were cost and quality-adjusted life-years (QALYs), which were both discounted at a rate of 5% [25]. Since China does not officially recommend willingness to pay (WTP), we used the WTP threshold from World Health Organization recommendation: We selected three times per capita gross domestic product (GDP) of China in 2021 ($35,424.12/QALY) for evaluating the incremental cost-effectiveness ratio (ICER) [26]. Moreover, half-cycle correction was performed for each cycle in the model.

### Treatment cost and utility

The health care perspective was selected and only direct treatment costs were considered in this study, involving immunotherapy and chemotherapy cost, adverse event cost, medical service cost, and follow up cost [27]. All costs were derived from the literature, and shown in 2022 US dollars (US $1 = CNY ¥6.8577) (Table 2).

**Table 2:** Input parameters in the partitioned survival model

	**Baseline value ($)**	**Range ($)**	**Distribution**	**Reference**
Durvalumab	7,866.6/ 1,500 mg	5,899.95–9,833.25	lognormal	[20]
Atezolizumab	4,755/1,200 mg	3,566.25–5,943.75	lognormal	[20]
Pembrolizumab	2,612.83/100 mg	1,959.69–3,266.11	lognormal	[27]
Nivolumab	665/40 mg	498.75–831.25	lognormal	[20]
Ipilimumab	81.2/mg	60.9–101.5	lognormal	[20]
Sintilimab	157.5/100 mg	118.13–196.88	lognormal	[28]
Camrelizumab	426.9/200 mg	320.2–533.7	lognormal	[29, 30]
Tislelizumab	317.89/100 mg	238.42–397.36	lognormal	[31]
Bevacizumab	262.5/100 mg	196.88–328.13	lognormal	[32]
Tremelimumab^a^	81.2/mg	60.9–101.5	lognormal	-
Carboplatin	0.16/mg	0.12–0.21	lognormal	[27]
Pemetrexed	131.24/100 mg	98.43–164.05	lognormal	[32]
Docetaxel	189.57/200 mg	142.18–236.96	lognormal	[33]
Cost of medical service of immunotherapy per cycle	8.20	6.15–10.25	lognormal	[27]
Cost of medical service of chemotherapy per cycle	45.97	34.48–57.47	lognormal	[27]
Follow-up cost per cycle	58.53	43.90–73.17	lognormal	[27, 34]
Utility for PFS	0.804	0.683–0.925	beta	[35]
Utility for PD	0.321	0.273–0.369	beta	[35]
Utility for death	0	0	-	-
Discount rate	5%	0–8%	-	[25]

^a^The price of tremelimumab was assumed to be identical to that of ipilimumab, because tremelimumab was not approved to enter into the market

(i) Drug cost: There was a similarity in the first-line drug regimens among 16 clinical trials (Table S2) [7–10, 23, 29, 36–45]. Thus, we set a unified but slightly different drug regimen (Table S3). At the time of disease progression, docetaxel was used in both the immunotherapy combination and chemotherapy group, according to subsequent treatment regimens shown in clinical trials [46].
(ii) Adverse event cost: Adverse events of grade ≥ 3 and occurred in ≥ 3% of cases were considered [7–10, 23, 29, 36–45]. We assumed that these adverse events occurred in the first cycle, because physicians might change treatment regimens if severe adverse events frequently occurred [27]. Owing to part of immunotherapy combinations (sint-chemo, pem-chemo, tisle-chemo, atezo-chemo) taken as intervention in more than one clinical trial, weighted adverse event incidence was used in our model (Table S4-S14).
(iii) Medical service cost: Fees of consultation, intravenous injection, nursing, and hospitalization were analysed in this study.
(iv) Follow up cost: Laboratory test and imaging examination involving urine tests, blood tests, blood biochemistry, and computed tomography were considered [27].

The utility of PFS, PD, and the main disutilities of adverse events for Chinese patients with NSCLC were derived from a utility research by Nafees [35].

### Sensitivity analyses

Deterministic sensitivity analyses (DSA) and probabilistic sensitivity analyses (PSA) were conducted to assess parameter uncertainty in model inputs. In DSA, 15% and 25% changes were assumed for utility and treatment cost [27, 47], respectively, while the discount rate was varied between 0 to 8% [25]. In PSA, lognormal distribution for cost, and beta distribution for utility were set for performing 1000 Monte Carlo simulation [48].

## Results

### Base-case results

Regarding the economic burden for patients, the incremental cost of camre-chemo versus chemo alone was $13,180.65, which was lowest among all other immunotherapy combinations. Sint-chemo was also found to be comparable to camre- chemo in treatment cost. Compared with single PD-1/PD-L1 inhibitor plus chemotherapy, dual drug combination led to a higher cost for patients. The incremental cost of atezo-beva-chemo, and nivo-ipi-chemo were $121,317.62, and $105,337.18, respectively (Table 3).

**Table 3:** Economic evaluation on immunotherapy combination versus chemotherapy

	**Cost ($)**	**Incremental Cost ($)**	**QALYs**	**Incremental QALYs**	**ICER ($/QALY)**
Chemo	19,505.60	-	0.93	-	-
Sint-chemo	35,216.04	15,710.44	1.38	0.45	34,912.09
Pem-chemo	102,747.84	83,242.24	1.35	0.42	198,195.81
Nivo-ipi-chemo	124,842.78	105,337.18	1.24	0.31	339,797.35
Tisle-chemo	37,738.81	18,233.21	1.28	0.35	52,094.89
Camre-chemo	32,686.25	13,180.65	1.22	0.29	45,450.52
Nivo-ipi	110,894.43	91,388.83	1.14	0.21	435,184.90
Atezo-beva-chemo	140,823.22	121,317.62	1.36	0.43	282,134.00
Durva-treme-chemo	112,804.77	93,299.17	1.05	0.12	777,493.08
Atezo-chemo	74,841.35	55,335.75	1.13	0.2	276,678.75
Durva-treme	73,073.61	53,568.01	0.91	-0.02	-

Regarding the health outcome, patients who experienced lower progression and death risk from immunotherapy combinations obtained greater QALY benefit. Among the immunotherapy combinations, sint-chemo provided the best QALY benefit versus chemo (incremental QALYs = 0.45), followed by atezo-beva-chemo (incremental QALYs = 0.43), and pem-chemo (incremental QALYs = 0.42).

In terms of the ICER, sint-chemo yielded the best ICER versus chemo (ICER = $34,912.09/QALY, under the WTP threshold), followed by camre-chemo (ICER = $45,450.52/QALY), and tisle-chemo (ICER = $52,094.89/QALY).

### Sensitivity analyses

**Fig. 2:**
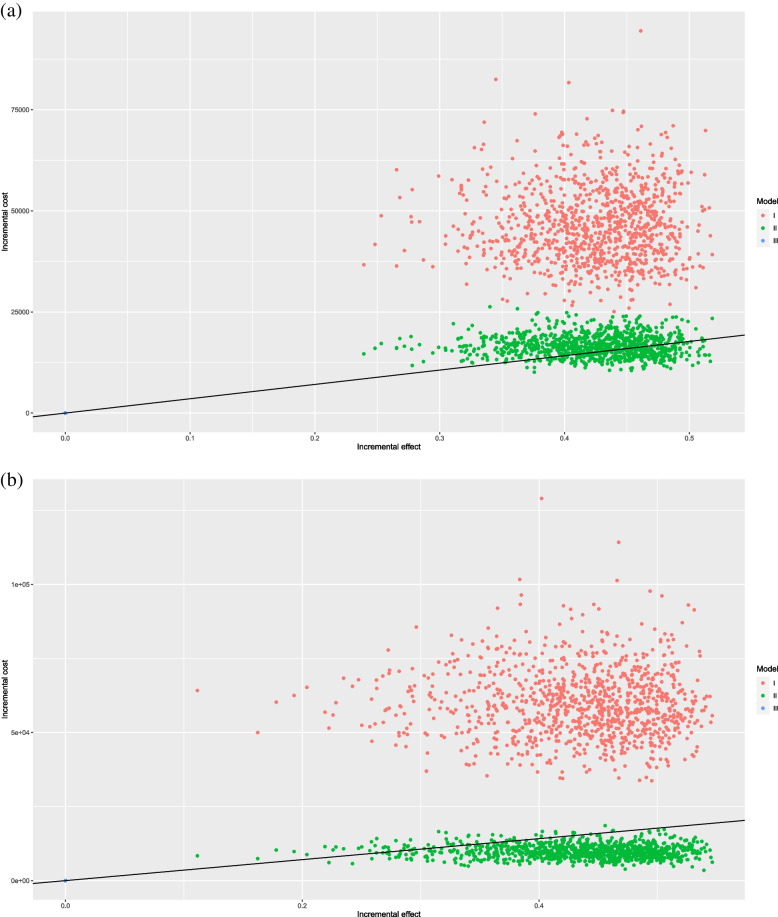
**a** Scatter diagram of pem-chemo versus chemo alone. I, pembrolizumab ($1,306.42/100 mg) plus chemotherapy; II, pembrolizumab ($261.28/100 mg) plus chemotherapy; III, chemotherapy alone. **b** Scatter diagram of atezo-beva-chemo versus chemo alone. I, atezolizumab ($2,377.5/1,200 mg) plus bevacizumab ($131.3/100 mg) plus chemotherapy; II, atezolizumab ($475.5/1200 mg) plus bevacizumab ($26.3/100 mg) plus chemotherapy; III, chemotherapy alone. WTP=$35,424.12

Tornado diagrams were presented to indicate that the drug price, PFS, and PD utilities, and discount rate were the primary factors influencing ICER (Figure S5-S14). The probabilistic sensitivity analyses were presented as scatter diagram (Figure S15-S24), which showed that the cost-effectiveness probability for sint-chemo was 56.88% (Figure S15). When the original price decreased by 90% for single PD-1 inhibitor-pembrolizumab, and dual drug combination-atezolizumab plus bevacizumab, the cost-effectiveness probabilities of these two immunotherapies were 32.01% and 93.91%, respectively (Fig. 2).

## Discussion

To our knowledge, our study is the first comprehensive economic evaluation for the main immunotherapy combinations. In the methodology, common patients from one trial (IMpower130) were explored to infer to other immunotherapy trials in order to adjust for the confounding factors. The ICERs of ten immunotherapy combinations compared with chemotherapy alone were from $34,912.09/QALY to $777,493.08/QALY. This finding serves as a basis for other developing countries (with per capital GDP and per capital health expenditure similar to that of China) to choose a reasonable and affordable price for health care system or payers.

Previous economic evaluation for head-to-head comparison of immunotherapy combination have shown that nivolumab plus ipilimumab was a cost-effective option in the United States, but not the preferred option in China [49, 50]. Moreover, pembrolizumab plus platinum and pemetrexed as the first-line treatment was not cost-effective in China [51]. Atezolizumab combination therapy was not cost-effective in the United States and China [52–54]. Our study revealed similar findings, and assessed more immune combination regimens.

The differences of ICER stemmed from the following reasons. First, transition probabilities in PFS, PD, and death were one of the main impact factors of ICER. HR was used to construct the PFS and OS curves of immunotherapy combinations and chemotherapy in this study. Thus, different HRs led a significant difference in the transition probability. For example, regarding OS, the HR of immunotherapy combination versus chemotherapy was from 0.59 to 0.94. Regarding PFS, this value was from 0.45 to 1.25 [22]. Second, the result of DSA showed that the utility of health state and the price of immunotherapy also contributed to these differences. The PFS and PD utility values were both obtained from a study conducted by Nafees. More comparisons and choices were limited due to the lack of evidence on the utility in the Chinese population. For the immunotherapy price, camrelizumab, toripalimab, and tislelizumab entered the national basic medical care insurance catalogue in China through price cut [31]. Although the price kept original such as pembrolizumab and nivolumab, new market strategies were conducted to be faced with the challenge from other PD-1 inhibitors. For example, the patient assistance program (PAP) of pembrolizumab was implemented: Patients who bought the first two cycles got the next two cycles for free, and then purchased the fifth and sixth cycles, the other cycles for two years were also free. The results of probabilistic sensitivity analysis also indicated that the cost-effectiveness probability for pem-chemo was 32.01% after the price of pembrolizumab dropped by 90%. Third, when PD-1/PD-L1 inhibitors combined with ipilimumab or bevacizumab, it increased the economic burden of patients.

The first PD-1 inhibitor-pembrolizumab was approved by US FDA in the year 2014. Accumulating evidence on immunotherapy from long-term follow-up studies and real-world data will render the HR more stable, thereby yielding more valid and reliable evidence on economic evaluation in the future. Meanwhile, there are controversies and various suggestions for the treatment of patients with NSCLC such as subsequent therapy for first-line receiving immunotherapy [34, 55]. The progress in these clinical areas will support specific and optimal choice for drug regimen set in the model. More importantly, the price strategy for acceptable ICER need both multinational pharmaceutical enterprises and Chinese local enterprises to maintain the balance between global pricing strategies and gaining more market share, single pricing strategy and various pricing strategies, the reimbursement of R&D cost and the affordability of patients, especially in the context of the global downward pressure on the economy.

This study has a few limitations. First, the treatment effect of durva-treme-chemo and atezo-beva-chemo respectively compared with chemotherapy alone only have indirect comparison in the network meta-analysis [22]. Although indirect comparison provide useful statistical technique to estimate treatment effect when direct comparisons are absent, they are low in power, with indeterminate results, and significant uncertainty [56–59]. The uncertainty of survival analysis might bring the uncertainty of economic evaluation in our study; therefore, head-to-head trial for durva-treme-chemo and atezo-beva-chemo respectively compared with chemotherapy alone would be warranted. Second, owing to lack of the network meta-analysis of each adverse event incidence [22], the weighted method was used in this study, thereby possibly increasing deviations compared with the synthetic evidence. Third, in the real-world, clinical treatments are complex and diverse. However, a unified drug regimen was set in order to highlight the differences among the immunotherapies. Fourth, the model in this study was based on a series of assumptions such as proportional hazard, which limited the extrapolation of the result.

## Conclusions

In the present study, we assessed the cost effectiveness of immunotherapy combinations versus chemotherapy alone in China. Among the immunotherapy combinations, sintilimab plus chemotherapy provided the best QALY benefit versus chemotherapy, and also appeared a better economic outcome. Although atezolizumab plus bevacizumab plus chemotherapy also provided favorable QALY, the economic outcome was unlikely to be ideal. Our findings revealed that much lower progression and death risk, and a competitive price for immunotherapy combination led to an acceptable ICER for Chinese patients with NSCLC. This serves as an evidence for pharmaceutical enterprises to properly and deeply consider the pricing strategy based on effectiveness and safety in the real-world condition.

### Supplementary Information

**Additional file 1: Table S1.** AIC and BIC of OS curve and PFS curve in the atezolizumab plus chemotherapy arm in the IMpower 130 trial. **Table S2.** Treatment regimens in 16 clinical trials. **Table S3.** Treatment regimens used in the partitioned survival model. **Table S4.** Serious adverse event cost and disutility for patients in China. **Table S5.** Serious adverse event incidence, cost and disutility of sint-chemo. **Table S6.** Serious adverse event incidence, cost and disutility of pem-chemo. **Table S7.** Serious adverse event incidence, cost and disutility of nivo-ipi-chemo. **Table S8.** Serious adverse event incidence, cost and disutility of tisle-chemo. **Table S9.** Serious adverse event incidence, cost and disutility of camre-chemo. **Table S10.** Serious adverse event incidence, cost and disutility of atezo-beva-chemo. **Table S11.** Serious adverse event incidence, cost and disutility of durva-treme-chemo. **Table S12.** Serious adverse event incidence, cost and disutility of atezo-chemo. **Table S13.** Serious adverse event incidence, cost and disutility of durva-treme. **Table S14.** Serious adverse event incidence, cost and disutility of chemo. **Figure S1.** Visual inspection of nine distributions for OS curve in the atezolizumab plus chemotherapy arm. **Figure S2.** Visual inspection of exponential, and loglogistic distributions for OS curve in the atezolizumab plus chemotherapy arm. **Figure S3.** Visual inspection of nine distributions for PFS curve in the atezolizumab plus chemotherapy arm. **Figure S4.** Visual inspection of genf, and loglogistic distributions for PFS curve in the atezolizumab plus chemotherapy arm. **Figure S5.** Deterministic sensitivity analysis of sint-chemo versus chemo. **FigureS6.** Deterministic sensitivity analysis of pem-chemo versus chemo. **Figure S7.** Deterministic sensitivity analysis of nivo-ipi-chemo versus chemo. **Figure S8.** Deterministic sensitivity analysis of tisle -chemo versus chemo. **Figure S9.** Deterministic sensitivity analysis of camre-chemo versus chemo. **Figure S10.** Deterministic sensitivity analysis of nivo-ipi versus chemo. **Figure S11.** Deterministic sensitivity analysis of atezo-beva-chemo versus chemo. **Figure S12.** Deterministic sensitivity analysis of durva-treme-chemo versus chemo. **Figure S13.** Deterministic sensitivity analysis of atezo-chemo versus chemo. **Figure S14.** Deterministic sensitivity analysis of durva-treme versus chemo. **Figure S15.** Probabilistic sensitivity analysis of sint-chemo versus chemo. **Figure S16.** Probabilistic sensitivity analysis of pem-chemo versus chemo. **Figure S17.** Probabilistic sensitivity analysis of nivo-ipi-chemo versus chemo. **Figure S18.** Probabilistic sensitivity analysis of tisle-chemo versus chemo. **Figure S19.** Probabilistic sensitivity analysis of camre-chemo versus chemo. **Figure S20.** Probabilistic sensitivity analysis of nivo-ipi versus chemo. **Figure S21.** Probabilistic sensitivity analysis of atezo-beva-chemo versus chemo. **Figure S22.** Probabilistic sensitivity analysis of durva-treme-chemo versus chemo. **Figure S23.** Probabilistic sensitivity analysis of atezo-chemo versus chemo. **Figure S24.** Probabilistic sensitivity analysis of durva-treme versus chemo.

## Data Availability

The datasets used during the study are available from the main text and supplementary material of this article.

## Abbreviations

NSCLC: Non-small cell lung cancer
HR: Hazard ratio
OS: Overall survival
PFS: Progression-free survival
PH: Proportional hazard
QALY: Quality-adjusted life-year
ICER: Incremental cost-effectiveness ratio
ICI: Immune checkpoint inhibitor
IPD: Individual patient data
AIC: Akaike information criterion
BIC: Bayesian information criterion
AFT: Accelerated failure time
WTP: Willingness to pay
GDP: Gross domestic product
DSA: Deterministic sensitivity analyse
PSA: Probabilistic sensitivity analyse
PAP: Patient assistance program
sint-chemo: Sintilimab plus chemotherapy
pem-chemo: Pembrolizumab plus chemotherapy
nivo-ipi-chemo: Nivolumab plus ipilimumab plus chemotherapy
tisle-chemo: Tislelizumab plus chemotherapy
camre-chemo: Camrelizumab plus chemotherapy
nivo-ipi: Nivolumab plus ipilimumab
atezo-beva-chemo: Atezolizumab plus bevacizumab plus chemotherapy
durva-treme-chemo: Durvalumab plus tremelimumab plus chemotherapy
atezo-chemo: Atezolizumab plus chemotherapy
durva-treme: Durvalumab plus tremelimumab
