# Supplementary material for: Cost-effectiveness of first-line immunotherapy combinations with or without chemotherapy for advanced non–small cell lung cancer: a modelling approach
Source: BMC Cancer. 2023 May 15;23:442. doi: 10.1186/s12885-023-10938-8 (PMC10186643; doi:10.1186/s12885-023-10938-8)
Supplement: Supplementary file 1 — Additional file 1: Table S1. AIC and BIC of OS curve and PFS curve in the atezolizumab plus chemotherapy arm in the IMpower 130 trial. Table S2. Treatment regimens in 16 clinical trials. Table S3. Treatment regimens used in the partitioned survival model. Table S4. Serious adverse event cost and disutility for patients in China. Table S5. Serious adverse event incidence, cost and disutility of sint-chemo. Table S6. Serious adverse event incidence, cost and disutility of pem-chemo. Table S7. Serious adverse event incidence, cost and disutility of nivo-ipi-chemo. Table S8. Serious adverse event incidence, cost and disutility of tisle-chemo. Table S9. Serious adverse event incidence, cost and disutility of camre-chemo. Table S10. Serious adverse event incidence, cost and disutility of atezo-beva-chemo. Table S11. Serious adverse event incidence, cost and disutility of durva-treme-chemo. Table S12. Serious adverse event incidence, cost and disutility of atezo-chemo. Table S13. Serious adverse event incidence, cost and disutility of durva-treme. Table S14. Serious adverse event incidence, cost and disutility of chemo. Figure S1. Visual inspection of nine distributions for OS curve in the atezolizumab plus chemotherapy arm. Figure S2. Visual inspection of exponential, and loglogistic distributions for OS curve in the atezolizumab plus chemotherapy arm. Figure S3. Visual inspection of nine distributions for PFS curve in the atezolizumab plus chemotherapy arm. Figure S4. Visual inspection of genf, and loglogistic distributions for PFS curve in the atezolizumab plus chemotherapy arm. Figure S5. Deterministic sensitivity analysis of sint-chemo versus chemo. FigureS6. Deterministic sensitivity analysis of pem-chemo versus chemo. Figure S7. Deterministic sensitivity analysis of nivo-ipi-chemo versus chemo. Figure S8. Deterministic sensitivity analysis of tisle -chemo versus chemo. Figure S9. Deterministic sensitivity analysis of camre-chemo versus chemo. Figure S10. Determinist [file 12885_2023_10938_MOESM1_ESM.docx]

**Supplementary material**

Table S1 AIC and BIC of OS curve and PFS curve in the atezolizumab plus chemotherapy arm in the IMpower 130 trial

Table S2 Treatment regimens in 16 clinical trials

Table S3 Treatment regimens used in the partitioned survival model

Table S4 Serious adverse event cost and disutility for patients in China

Table S5 Serious adverse event incidence, cost and disutility of sint-chemo

Table S6 Serious adverse event incidence, cost and disutility of pem-chemo

Table S7 Serious adverse event incidence, cost and disutility of nivo-ipi-chemo

Table S8 Serious adverse event incidence, cost and disutility of tisle-chemo

Table S9 Serious adverse event incidence, cost and disutility of camre-chemo

Table S10 Serious adverse event incidence, cost and disutility of atezo-beva-chemo

Table S11 Serious adverse event incidence, cost and disutility of durva-treme-chemo

Table S12 Serious adverse event incidence, cost and disutility of atezo-chemo

Table S13 Serious adverse event incidence, cost and disutility of durva-treme

Table S14 Serious adverse event incidence, cost and disutility of chemo

Figure S1 Visual inspection of nine distributions for OS curve in the atezolizumab plus chemotherapy arm

Figure S2 Visual inspection of exponential, and loglogistic distributions for OS curve in the atezolizumab plus chemotherapy arm

Figure S3 Visual inspection of nine distributions for PFS curve in the atezolizumab plus chemotherapy arm

Figure S4 Visual inspection of genf, and loglogistic distributions for PFS curve in the atezolizumab plus chemotherapy arm

Figure S5 Deterministic sensitivity analysis of sint-chemo versus chemo

FigureS6 Deterministic sensitivity analysis of pem-chemo versus chemo

Figure S7 Deterministic sensitivity analysis of nivo-ipi-chemo versus chemo

Figure S8 Deterministic sensitivity analysis of tisle -chemo versus chemo

Figure S9 Deterministic sensitivity analysis of camre-chemo versus chemo

Figure S10 Deterministic sensitivity analysis of nivo-ipi versus chemo

Figure S11 Deterministic sensitivity analysis of atezo-beva-chemo versus chemo

Figure S12 Deterministic sensitivity analysis of durva-treme-chemo versus chemo

Figure S13 Deterministic sensitivity analysis of atezo-chemo versus chemo

Figure S14 Deterministic sensitivity analysis of durva-treme versus chemo

Figure S15 Probabilistic sensitivity analysis of sint-chemo versus chemo

Figure S16 Probabilistic sensitivity analysis of pem-chemo versus chemo

Figure S17 Probabilistic sensitivity analysis of nivo-ipi-chemo versus chemo

Figure S18 Probabilistic sensitivity analysis of tisle-chemo versus chemo

Figure S19 Probabilistic sensitivity analysis of camre-chemo versus chemo

Figure S20 Probabilistic sensitivity analysis of nivo-ipi versus chemo

Figure S21 Probabilistic sensitivity analysis of atezo-beva-chemo versus chemo

Figure S22 Probabilistic sensitivity analysis of durva-treme-chemo versus chemo

Figure S23 Probabilistic sensitivity analysis of atezo-chemo versus chemo

Figure S24 Probabilistic sensitivity analysis of durva-treme versus chemo

**Article title:** Cost-effectiveness of first-line immunotherapy combinations with or without chemotherapy for advanced non–small cell lung cancer: a modelling approach

**Journal name:** BMC Cancer

**Corresponding author:** Yuanyi Cai, PHD: Department of Health Service Management, School of Health Management, China Medical University, Shenyang, China

Email: yycai@cmu.edu.cn

**Table S1 AIC and BIC of OS curve and PFS curve in the atezolizumab plus chemotherapy arm in the IMpower 130 trial**

| **Distribution** | **OS curve** | | **PFS curve** | |
| --- | --- | --- | --- | --- |
|  | **AIC** | **BIC** | **AIC** | **BIC** |
| Exponential | 1988.973 | 1993.084 | 2329.778 | 2333.889 |
| Gamma | 1986.720 | 1994.943 | 2314.370 | 2322.593 |
| Genf | 1988.058 | 2004.504 | 2292.443 | 2308.888 |
| Gengamma | 1986.056 | 1998.390 | 2300.086 | 2312.421 |
| Gompertz | 1990.353 | 1998.575 | 2331.633 | 2339.856 |
| Weibull | 1987.585 | 1995.808 | 2320.647 | 2328.870 |
| WeibullPH | 1987.585 | 1995.808 | 2320.647 | 2328.870 |
| Loglogistic | 1984.869 | 1993.092 | 2294.280 | 2302.503 |
| Lognormal | 1987.992 | 1996.215 | 2300.034 | 2308.257 |

**Table S2 Treatment regimens in 16 clinical trials**

|  | **Induction therapy** | **Maintenance therapy** | **Immunotherapy period** | **Subsequent therapy** |
| --- | --- | --- | --- | --- |
| KEYNOTE-021G | Arm1:Pembrolizumab 200 mg Q3W + chemotherapy (carboplatin AUC 5 + pemetrexed 500 mg/m^2^ Q3W) for 4 cycles  Arm2: Chemotherapy (carboplatin AUC 5 + pemetrexed 500 mg/m^2^ Q3W) for 4 cycles | Arm1: Pembrolizumab 200 mg + pemetrexed 500 mg/m^2^ Q3W  Arm2:Pemetrexed 500 mg/m^2^ Q3W | 2 years | Arm1: Not mentioned  Arm2: Not mentioned |
| KEYNOTE-189 | Arm1: Pembrolizumab 200 mg Q3W + chemotherapy (cisplatin 75 mg/m^2^ or carboplatin AUC 5 Q3W + pemetrexed 500 mg/m^2^) for 4 cycles  Arm2: Saline placebo + chemotherapy (cisplatin 75 mg/m^2^ or carboplatin AUC 5 on Q3W + pemetrexed 500 mg/m^2^) for 4 cycles | Arm1: Pembrolizumab 200 mg + pemetrexed 500 mg/m^2^ Q3W  Arm2: Saline placebo + pemetrexed 500 mg/m^2^ Q3W | 2 years | Arm1:13.4% PD-1 or PD-L1 inhibitor, 31.2% other therapies  Arm2:40.8%pembrolizumab,13.1% other PD-1 or PD-L1 inhibitors, 5.3% other therapies |
| KEYNOTE-407 | Arm1: Pembrolizumab 200 mg Q3W + chemotherapy(carboplatin AUC 6 + paclitaxel 200 mg/m^2^ or nab-paclitaxel 100 mg/m^2^ Q3W) for 4 cycles  Arm2:Saline placebo + chemotherapy(carboplatin AUC 6 + paclitaxel 200 mg/m^2^ or nab-paclitaxel 100 mg/m^2^ Q3W) for 4 cycles | Arm1: Pembrolizumab 200 mg Q3W  Arm2: Saline placebo Q3W | 2 years | Arm1: Not mentioned  Arm2: 40.1% pembrolizumab,  9% other PD-1 or PD-L1 inhibitors  10.3% other therapies |

**Table S2 Continued**

|  | **Induction therapy** | **Maintenance therapy** | **Immunotherapy period** | **Subsequent therapy** |
| --- | --- | --- | --- | --- |
| IMpower-130 | Arm1: Atezolizumab 1200 mg Q3W + chemotherapy (carboplatin AUC 6 Q3W + nab-paclitaxel 100 mg/m^2^ QW) for 4 or 6 cycles  Arm2: Chemotherapy (carboplatin AUC 6 Q3W + nab-paclitaxel 100 mg/m^2^ QW) for 4 or 6 cycles | Arm1: Atezolizumab 1200 mg  Arm2: Best supportive care or pemetrexed | Not mentioned | Arm1: Not mentioned  Arm2: Not mentioned |
| IMpower-131 | Arm1: Atezolizumab 1200 mg Q3W+ chemotherapy (carboplatin AUC 6 Q3W+ paclitaxel 200 mg/m^2^ Q3W) for 4 or 6 cycles  Arm2: Atezolizumab 1200 mg Q3W + chemotherapy (carboplatin AUC 6 Q3W+ nab-paclitaxel 100 mg/m^2^ Q3W) for 4 or 6 cycles  Arm3: Chemotherapy (carboplatin AUC 6 Q3W+ nab-paclitaxel 100 mg/m^2^ Q3W) for 4 or 6 cycles | Arm1:Atezolizumab 1200 mg  Arm2:Atezolizumab 1200 mg  Arm3: Not mentioned | Not mentioned | Arm1: 7.1% immunotherapy  29.6% chemotherapy  7.1% targeted therapy  Arm2: 6.4% immunotherapy  32.1% chemotherapy  7.3% targeted therapy  Arm3: 43.2% immunotherapy  27.4% chemotherapy  5.9% targeted therapy |
| IMpower-132 | Arm1: Atezolizumab 1200 mg Q3W + Chemotherapy (carboplatin AUC 6 or cisplatin 75 mg/m^2^ +pemetrexed 500 mg/m^2^ Q3W) for 4 to 6 cycles  Arm2: Chemotherapy (carboplatin AUC 6 or cisplatin 75 mg/m^2^ +pemetrexed 500 mg/m^2^ Q3W) for 4 to 6 cycles | Arm1: Atezolizumab1200 mg+ pemetrexed 500 mg/m^2;^  Arm2: Pemetrexed 500 mg/m^2^ | Not mentioned | Arm1: 5.5% immunotherapy  34.9% chemotherapy  15.8% targeted therapy  Arm2: 45.8% immunotherapy  29% chemotherapy  14.3% targeted therapy |

**Table S2 Continued**

|  | **Induction therapy** | **Maintenance therapy** | **Immunotherapy period** | **Subsequent therapy** |
| --- | --- | --- | --- | --- |
| IMpower-150 | Arm1: Atezolizumab 1200 mg Q3W + bevacizumab 15 mg/ kg Q3W + chemotherapy [ carboplatin AUC6 Q3W+ paclitaxel 200 mg/m^2^ (175 mg/m^2^ for Asian patients)] 4 or 6 cycles  Arm2: Atezolizumab 1200 mg Q3W + chemotherapy [ carboplatin AUC6 Q3W+ paclitaxel 200 mg/m^2^ (175 mg/m^2^ for Asian patients)] 4 or 6 cycles  Arm3: Bevacizumab 15 mg/ kg Q3W + chemotherapy [ carboplatin AUC6 Q3W+ paclitaxel 200 mg/m^2^ (175 mg/m^2^ for Asian patients)] 4 or 6 cycles | Arm1: Atezolizumab 1200 mg + bevacizumab 15 mg/ kg  Arm2: Atezolizumab 1200 mg  Arm3: Bevacizumab 15 mg/ kg | Not mentioned | Arm1: Not mentioned  Arm2: Not mentioned  Arm3: Not mentioned |
| CheckMate-9LA | Arm1: Nivolumab 360 mg Q3W + ipilimumab 1 mg/kg Q6W +chemotherapy (carboplatin AUC 6 or cisplatin 75 mg/m^2^ + pemetrexed 500 mg/m^2^ Q3W) for 2 cycles  Arm2: Chemotherapy (carboplatin AUC 6or cisplatin 75 mg/m^2^ + pemetrexed 500 mg/m^2^ Q3W) for 4 cycles | Arm1:Nivolumab 360 mg + ipilimumab 1 mg/kg  Arm2: Pemetrexed 500 mg/m^2^ Q3W for patients with nonsquamous histology | 2 years | Arm1:15% radiotherapy, 7% immunotherapy, 6% targeted therapy, 32% chemotherapy, 18% platinum-doublet chemotherapy  Arm2: 15% radiotherapy, 36% immunotherapy, 8% targeted therapy, 24% chemotherapy, 5% platinum-doublet chemotherapy |

**Table S2 Continued**

|  | **Induction therapy** | **Maintenance therapy** | **Immunotherapy period** | **Subsequent therapy** |
| --- | --- | --- | --- | --- |
| CheckMate-227  Part 1 | Arm1: Nivolumab 3 mg/kg Q2W + ipilimumab 1mg/kg Q6W  Arm2: Nivolumab 240mg Q2W  Arm3:Platinum doublet chemotherapy (carboplatin AUC 6 or cisplatin 75 mg/m^2^ +pemetrexed 500 mg/m^2^ Q3W) for 4 cycles | Arm1:Nivolumab 3 mg/kg + ipilimumab 1mg/kg  Arm2: Nivolumab 240mg  Arm3: Pemetrexed 500 mg/m^2^ Q3W for patients with nonsquamous histology | 2 years | Arm1: 18.2% radiotherapy, 2.3% surgery, 35.9% chemotherapy, 7.3% immunotherapy, 7.3% targeted therapy, 1.8% experimental drugs  Arm3: 19.2% radiotherapy, 2.7% surgery, 27.5% chemotherapy, 37.5% immunotherapy, 10.2% targeted therapy, 2.7% experimental drugs |
| MYSTIC | Arm1: Durvalumab 20 mg/kg+tremelimumab 1 mg/kg Q4W  Arm2: Durvalumab 20 mg/kg Q4W  Arm3: Chemotherapy (carboplatin AUC 6 or cisplatin 75 mg/m^2^ + pemetrexed 500 mg/m^2^ Q3W) for 4 to 6 cycles | Arm1:Durvalumab 20 mg/kg  Arm2:Durvalumab 20 mg/kg  Arm3: Pemetrexed 500 mg/m^2^ for patients with nonsquamous NSCLC | Not mentioned | Arm1: 3.1% immunotherapy, 31.9% chemotherapy, 5.5% other systemic therapies  Arm2: 6.1% immunotherapy, 42.9% chemotherapy, 11% other systemic therapies  Arm3: 39.5% immunotherapy, 35.8% chemotherapy, 11.1% other systemic therapies |
| CameL | Arm1: Camrelizumab 200mg Q3W + chemotherapy (carboplatin AUC 5 + pemetrexed 500 mg/m^2^ Q3W) for 4 to 6 cycles  Arm2:Chemotherapy (carboplatin AUC 5+pemetrexed 500 mg/m^2^ Q3W) for 4 to 6 cycles | Arm1: Camrelizumab 200 mg + pemetrexed 500 mg/m^2^ Q3W  Arm2: Pemetrexed 500 mg/m^2^ Q3W | 2 years | Arm1: Not mentioned  Arm2: Not mentioned |

**Table S2 Continued**

|  | **Induction therapy** | **Maintenance therapy** | **Immunotherapy period** | **Subsequent therapy** |
| --- | --- | --- | --- | --- |
| CCTG BR-34 | Arm1: Durvalumab 1500 mg Q3W + tremelimumab 75 mg Q3W + chemotherapy (carboplatin AUC 6 or cisplatin 75 mg/m^2^ + pemetrexed 500 mg/m^2^ Q3W) for 4 cycles  Arm2: Durvalumab 1500 mg Q4W + tremelimumab 75 mg Q4W for 4 cycles | Arm1：Durvalumab 1500 mg Q4W + pemetrexed 500 mg/m^2^ Q4W for patients with nonsquamous histology  Arm2: Durvalumab 1500 mg | Not mentioned | Arm1: 39% radiotherapy, 1% surgery, 3% platinum-doublet, 10% docetaxel, 1% nivolumab/ pembrolizumab  Arm2: 38% radiotherapy, 1% surgery, 42% platinum-doublet, 2% docetaxel, 2% afatinib, 3% nivolumab/ pembrolizumab |
| RATIONALE-304 | Arm1: Tislelizumab 200 mg + chemotherapy (carboplatin AUC 5 or cisplatin 75 mg/m^2^ + pemetrexed 500 mg/m^2^ Q3W) for 4 to 6 cycles  Arm2: Chemotherapy (carboplatin AUC 5 or cisplatin 75 mg/m^2^ + pemetrexed 500 mg/m^2^ Q3W) for 4 to 6 cycles | Arm1: Tislelizumab 200 mg + pemetrexed 500 mg/m^2^  Arm2: Pemetrexed 500 mg/m^2^ Q3W | Not mentioned | Arm1: Not mentioned  Arm2: Not mentioned |
| RATIONALE-307 | Arm 1: Tislelizumab 200mg + chemotherapy (carboplatin AUC 5 +paclitaxel 175 mg/m^2^ Q3W)  Arm2：Tislelizumab 200mg + chemotherapy (carboplatin AUC 5 + nab-paclitaxel 100 mg/m^2^ Q3W)  Arm3： Chemotherapy (carboplatin AUC 5 +paclitaxel 175 mg/m^2^ Q3W) for 4 to 6 cycles | Arm1:Tislelizumab 200mg Q3W  Arm2:Tislelizumab 200mg Q3W  Arm3: Not mentioned | Not mentioned | Arm1: Not mentioned  Arm2: Not mentioned  Arm3: Not mentioned |

**Table S2 Continued**

|  | **Induction therapy** | **Maintenance therapy** | **Immunotherapy period** | **Subsequent therapy** |
| --- | --- | --- | --- | --- |
| ORIENT-11 | Arm1：Sintilimab 200 mg Q3W + chemotherapy (carboplatin AUC 5 or cisplatin 75 mg/m^2^ + pemetrexed 500 mg/m^2^ Q3W) for 4 cycles  Arm2:Placebo + Chemotherapy (carboplatin AUC 5 or cisplatin 75 mg/m^2^ + pemetrexed 500 mg/m^2^ Q3W) for 4 cycles | Arm1: Sintilimab 200 mg + pemetrexed 500 mg/m^2^ Q3W  Arm2: Placebo + pemetrexed 500 mg/m^2^ Q3W | 2 years | Arm1: Not mentioned  Arm2: Not mentioned |
| ORIENT-12 | Arm1：Sintilimab 200 mg + chemotherapy (carboplatin AUC 5 or cisplatin 75 mg/m^2^+gemcitabine 1 g/m^2^) Q3W for 4 or 6 cycles  Arm2：Placebo +Chemotherapy (gemcitabine 1 g/m^2^ + carboplatin AUC 5 or cisplatin 75 mg/m^2^) Q3W for 4 or 6 cycles | Arm1: Sintilimab 200 mg Q3W  Arm2: Placebo Q3W | 2 years | Arm1: Not mentioned  Arm2: Not mentioned |

**Ta****ble S3 Treatment regimens used in the partitioned survival model**

|  | **Immunotherapy combination group** | **Chemotherapy group** |
| --- | --- | --- |
| Progression-free survival |  |  |
| The first two or four cycles | Immunotherapy+Carboplatin+Pemetrexed or Immunotherapy | Carboplatin+Pemetrexed |
| The 3rd or 5th cycle to 2 years | Immunotherapy+Pemetrexed or Immunotherapy | Pemetrexed |
| Subsequent therapy | Pemetrexed | Pemetrexed |
| Progressive disease | Docetaxel | Docetaxel |

**Table S4 Serious adverse event cost and disutility for patients in China**

|  | **Medical cost ($)** | **Reference** | **Disutility per event** | **Reference** |
| --- | --- | --- | --- | --- |
| Anemia | 570.07 | [1] | 0.09 | [7] |
| Neutropenia^a^ | 511.38 | [2] | 0.2 | [8] |
| Leukopenia^b^ | 1143.97 | [3] | 0.2 | [8] |
| Thrombocytopenia^c^ | 519.93 | [1] | 0.2 | [8] |
| Nausea^d^ | 13.79 | [2] | 0.12 | [8] |
| Fatigue^e^ | 127.89 | [2] | 0.07 | [8] |
| Diarrhea | 5.57 | [1] | 0.07 | [8] |
| Increased alanine aminotransferase^h^ | 24.6 | [4] | 0.19 | [8] |
| Rash | 5.58 | [2] | 0.1 | [8] |
| Gamma-glutamyltransferase increased^i^ | 24.6 | [4] | 0.19 | [8] |
| Hypertension | 12.25 | [5] | 0.04 | [8] |
| Febrile neutropenia | 958.6 | [6] | 0.42 | [8] |
| Infectious pneumonitis | 1066.54 | [3] | 0.09 | [9] |
| Hyponatremia^f,j^ | 5.57 | [1] | 0.19 | [8] |
| Dyspnea^k^ | 1066.54 | [3] | 0.09 | [9] |
| Decreased appetite^g,l^ | 13.79 | [2] | 0.12 | [8] |

^a^Neutropenia included reports of decreased neutrophil count and neutropenia.

^b^Leukopenia included reports of decreased white blood cell count and leucopenia.

^c^Thrombocytopenia included reports of decreased platelet count and thrombocytopenia.

^d^Nausea included reports of vomiting and nausea.

^e^Fatigue included reports of asthenia, fatigue, and malaise.

^f^The medical cost of hyponatremia was assumed to be identical to that of diarrhea.

^g^The medical cost of decreased appetite was assumed to be identical to that of nausea.

^h^The disutility of increased alanine aminotransferase was assumed to be the disutility of nausea plus fatigue.

^i^The disutility of gamma-glutamyltransferase increased was assumed to be the disutility of nausea plus fatigue.

^j^The disutility of hyponatremia was assumed to be the disutility of nausea plus fatigue.

^k^The disutility of dyspnea was assumed to be identical to that of infectious pneumonitis.

^l^The disutility of decreased appetite was assumed to be identical to that of nausea.

**Table S5 Serious adverse event incidence, cost and disutility of sint-chemo^a^**

|  | **Grade ≥3** | **Medical cost ($)** | **SAE costs ($)** | **Disutility per event** | **SAE Disutility** |
| --- | --- | --- | --- | --- | --- |
| Anemia | 22.44% | 570.07 | 127.93 | 0.09 | 0.020 |
| Neutropenia | 41.37% | 511.38 | 211.54 | 0.2 | 0.083 |
| Leukopenia | 23.39% | 1143.97 | 267.56 | 0.2 | 0.047 |
| Thrombocytopenia | 25.39% | 519.93 | 132.04 | 0.2 | 0.051 |
| Infectious pneumonitis | 14% | 1066.54 | 149.32 | 0.09 | 0.013 |
| Hyponatremia | 6.10% | 5.57 | 0.34 | 0.19 | 0.012 |

Abbreviation: SAE: serious adverse event; sint-chemo: sintilimab plus chemotherapy

^a^Sint-chemo was taken as intervention arm in two trials including ORIENT-11 and ORIENT-12. The incidence of serious adverse event for sint-chemo in this study was weighted by the sample size from the two trials above.

**Table S6 Serious adverse event incidence, cost and disutility of pem-chemo^a^**

|  | **Grade ≥3** | **Medical cost ($)** | **SAE costs ($)** | **Disutility per event** | **SAE Disutility** |
| --- | --- | --- | --- | --- | --- |
| Anemia | 16.86% | 570.07 | 96.13 | 0.09 | 0.015 |
| Neutropenia | 17.91% | 511.38 | 91.57 | 0.2 | 0.036 |
| Thrombocytopenia | 8.36% | 519.93 | 43.46 | 0.2 | 0.017 |
| Fatigue | 9.42% | 127.89 | 12.05 | 0.07 | 0.007 |
| Diarrhea | 4.83% | 5.57 | 0.27 | 0.07 | 0.003 |
| Dyspnea | 4.20% | 1066.54 | 44.79 | 0.09 | 0.004 |

Abbreviation: SAE: serious adverse event; pem-chemo: pembrolizumab plus chemotherapy

^a^Pem-chemo was taken as intervention arm in three trials including KEYNOTE-021, KEYNOTE-189, and KEYNOTE-407. The incidence of serious adverse event for pem-chemo in this study was weighted by the sample size from the three trials above.

**Table S7 Serious adverse event incidence, cost and disutility of nivo-ipi-chemo^a^**

|  | **Grade ≥3** | **Medical cost ($)** | **SAE costs ($)** | **Disutility per event** | **SAE Disutility** |
| --- | --- | --- | --- | --- | --- |
| Anemia | 6% | 570.07 | 34.20 | 0.09 | 0.005 |
| Neutropenia | 7% | 511.38 | 35.80 | 0.2 | 0.014 |
| Diarrhea | 4% | 5.57 | 0.22 | 0.07 | 0.003 |

Abbreviation: SAE: serious adverse event; nivo-ipi-chemo: nivolumab plus ipilimumab plus chemotherapy

^a^ Another trial involving nivo was checkMate-227 trial. There were no adverse events of grade greater than or equal to 3 and occurred in ≥3% in the nivo-ipi arm in this trial.

**Table S8 Serious adverse event incidence, cost and disutility of tisle-chemo^a^**

|  | **Grade ≥3** | **Medical cost ($)** | **SAE costs ($)** | **Disutility per event** | **SAE Disutility** |
| --- | --- | --- | --- | --- | --- |
| Anemia | 15.02% | 570.07 | 85.63 | 0.09 | 0.014 |
| Neutropenia | 62.40% | 511.38 | 319.09 | 0.2 | 0.125 |
| Leukopenia | 33.88% | 1143.97 | 387.61 | 0.2 | 0.068 |
| Thrombocytopenia | 18.72% | 519.93 | 97.32 | 0.2 | 0.037 |

Abbreviation: SAE: serious adverse event; tisle-chemo: tislelizumab plus chemotherapy

^a^Tisle-chemo was taken as intervention arm in two trials including RATIONALE-304 and RATIONALE-307. The incidence of serious adverse event for tisle -chemo in this study was weighted by the sample size from the two trials above.

**Table S9 Serious adverse event incidence, cost and disutility of camre-chemo**

|  | **Grade ≥3** | **Medical cost ($)** | **SAE costs ($)** | **Disutility per event** | **SAE Disutility** |
| --- | --- | --- | --- | --- | --- |
| Anemia | 19% | 570.07 | 108.31 | 0.09 | 0.017 |
| Neutropenia | 38% | 511.38 | 194.32 | 0.2 | 0.076 |
| Leukopenia | 24% | 1143.97 | 274.55 | 0.2 | 0.048 |
| Thrombocytopenia | 17% | 519.93 | 88.39 | 0.2 | 0.034 |
| Fatigue | 3% | 127.89 | 3.84 | 0.07 | 0.002 |
| Increased alanine aminotransferase | 5% | 24.6 | 1.23 | 0.19 | 0.010 |
| Gamma-glutamyltransferase increased | 3% | 24.6 | 0.74 | 0.19 | 0.006 |

Abbreviation: SAE: serious adverse event; camre-chemo: camrelizumab plus chemotherapy

**Table S10 Serious adverse event incidence, cost and disutility of atezo-beva-chemo**

|  | **Grade ≥3** | **Medical cost ($)** | **SAE costs ($)** | **Disutility per event** | **SAE Disutility** |
| --- | --- | --- | --- | --- | --- |
| Anemia | 6.10% | 570.07 | 34.77 | 0.09 | 0.005 |
| Neutropenia | 22.40% | 511.38 | 114.55 | 0.2 | 0.045 |
| Thrombocytopenia | 9.20% | 519.93 | 47.83 | 0.2 | 0.018 |
| Nausea | 3.80% | 13.79 | 0.52 | 0.12 | 0.005 |
| Fatigue | 3.30% | 127.89 | 4.22 | 0.07 | 0.002 |
| Hypertension | 6.40% | 12.25 | 0.78 | 0.04 | 0.003 |
| Febrile neutropenia | 8.40% | 958.6 | 80.52 | 0.42 | 0.035 |

Abbreviation: SAE: serious adverse event; atezo-beva-chemo: atezolizumab plus bevacizumab plus chemotherapy

**Table S11 Serious adverse event incidence, cost and disutility of durva-treme-chemo**

|  | **Grade ≥3** | **Medical cost ($)** | **SAE costs ($)** | **Disutility per event** | **SAE Disutility** |
| --- | --- | --- | --- | --- | --- |
| Anemia | 24% | 570.07 | 136.82 | 0.09 | 0.022 |
| Neutropenia | 36% | 511.38 | 184.10 | 0.2 | 0.072 |
| Leukopenia | 26% | 1143.97 | 297.43 | 0.2 | 0.052 |
| Thrombocytopenia | 18% | 519.93 | 93.59 | 0.2 | 0.036 |
| Nausea | 7% | 13.79 | 0.97 | 0.12 | 0.008 |
| Fatigue | 5% | 127.89 | 6.39 | 0.07 | 0.004 |
| Diarrhea | 5% | 5.57 | 0.28 | 0.07 | 0.004 |
| Rash | 5% | 5.58 | 0.28 | 0.1 | 0.005 |

Abbreviation: SAE: serious adverse event; durva-treme-chemo: durvalumab plus tremelimumab plus chemotherapy

**Table S12 Serious adverse event incidence, cost and disutility of atezo-chemo^a^**

|  | **Grade ≥3** | **Medical cost ($)** | **SAE costs ($)** | **Disutility per event** | **SAE Disutility** |
| --- | --- | --- | --- | --- | --- |
| Anemia | 26.19% | 570.07 | 149.28 | 0.09 | 0.024 |
| Neutropenia | 39.07% | 511.38 | 199.82 | 0.2 | 0.078 |
| Leukopenia | 7.74% | 1143.97 | 88.52 | 0.2 | 0.015 |
| Thrombocytopenia | 15.68% | 519.93 | 81.50 | 0.2 | 0.031 |
| Fatigue | 5.13% | 127.89 | 6.56 | 0.07 | 0.004 |
| Diarrhea | 4.17% | 5.57 | 0.23 | 0.07 | 0.003 |
| Decreased appetite | 3% | 13.79 | 0.41 | 0.12 | 0.004 |

Abbreviation: SAE: serious adverse event; ate-chemo: atezolizumab plus chemotherapy

^a^atezo-chemo was taken as intervention arm in three trials including IMpower-130, IMpower-131, and IMpower-132. The incidence of serious adverse event for atezolizumab -chemo in this study was weighted by the sample size from the three trials above.

**Table S13 Serious adverse event incidence, cost and disutility of durva-treme**

|  | **Grade ≥3** | **Medical cost ($)** | **SAE costs ($)** | **Disutility per event** | **SAE Disutility** |
| --- | --- | --- | --- | --- | --- |
| Fatigue | 3% | 127.89 | 3.84 | 0.07 | 0.002 |
| Diarrhea | 3% | 5.57 | 0.17 | 0.07 | 0.002 |
| Dyspnea | 4.2% | 1066.54 | 44.79 | 0.09 | 0.004 |

Abbreviation: SAE: serious adverse event; durva-treme: durvalumab plus tremelimumab

**Table S14 Serious adverse event incidence, cost and disutility of chemo^a^**

|  | **Grade ≥3** | **Medical cost ($)** | **SAE costs ($)** | **Disutility per event** | **SAE Disutility** |
| --- | --- | --- | --- | --- | --- |
| Anemia | 15.05% | 570.07 | 85.80 | 0.09 | 0.014 |
| Neutropenia | 24.27% | 511.38 | 124.09 | 0.2 | 0.049 |
| Leukopenia | 16.34% | 1143.97 | 186.90 | 0.2 | 0.033 |
| Thrombocytopenia | 9.74% | 519.93 | 50.65 | 0.2 | 0.019 |
| Fatigue | 5.91% | 127.89 | 7.56 | 0.07 | 0.004 |
| Diarrhea | 4.07% | 5.57 | 0.23 | 0.07 | 0.003 |

Abbreviation: SAE: serious adverse event; chemo: chemotherapy

^a^chemo was taken as control arm in fifteen trials including ORIENT-11, ORIENT-12, KEYNOTE-021, KEYNOTE-189, KEYNOTE-407, CheckMate-9LA, RATIONALE-304, RATIONALE-307, CameL, CheckMate-227, IMpower-150, IMpower-130, IMpower-131, IMpower-132, and MYSTIC. The incidence of serious adverse event for chemo in this study was weighted by the sample size from the fifteen trials above.


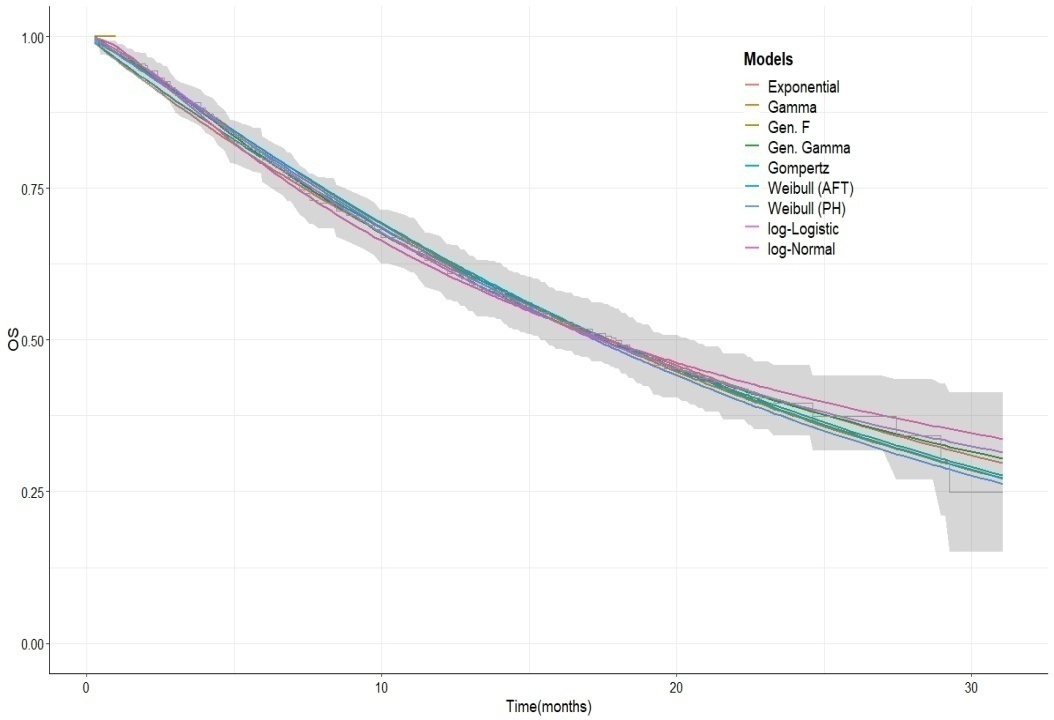


**Figure S1** Visual inspection of nine distributions for OS curve in the atezolizumab plus chemotherapy arm


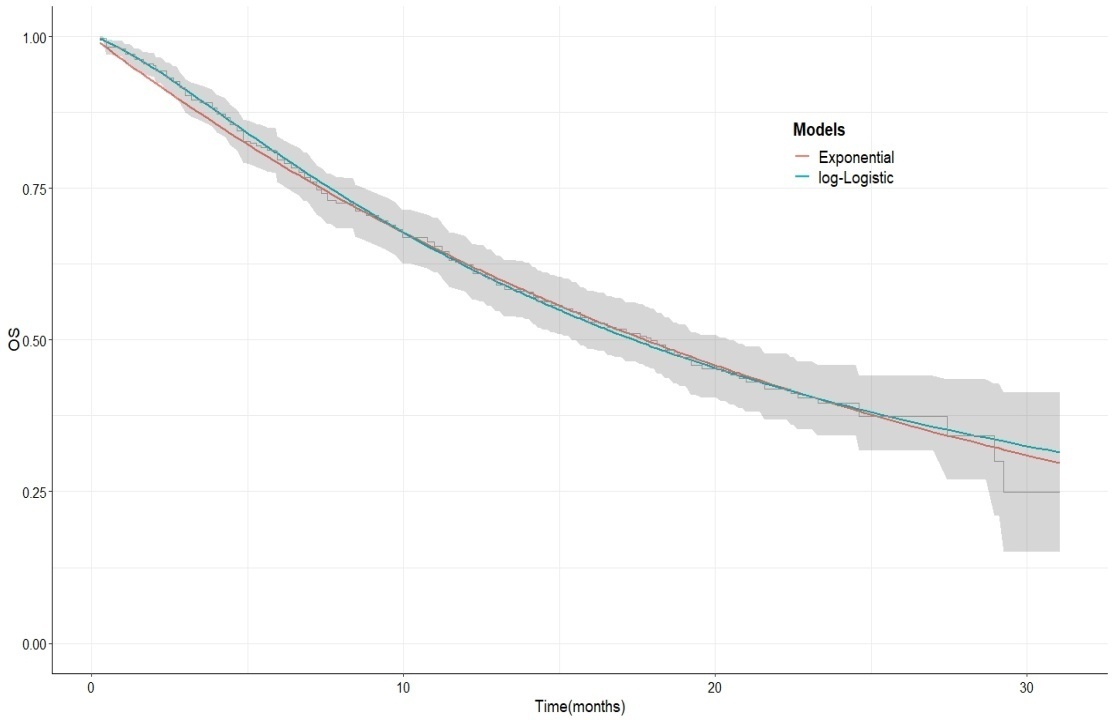


**Figure S2** Visual inspection of exponential, and loglogistic distributions for OS curve in the atezolizumab plus chemotherapy arm


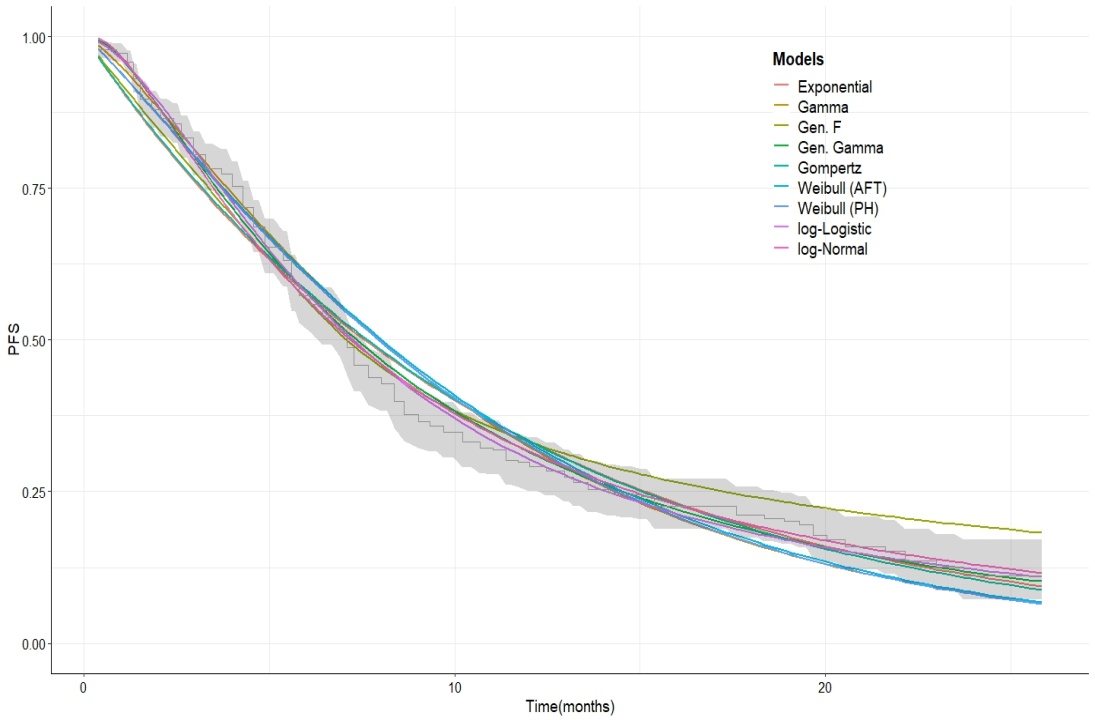


**Figure S3** Visual inspection of nine distributions for PFS curve in the atezolizumab plus chemotherapy arm


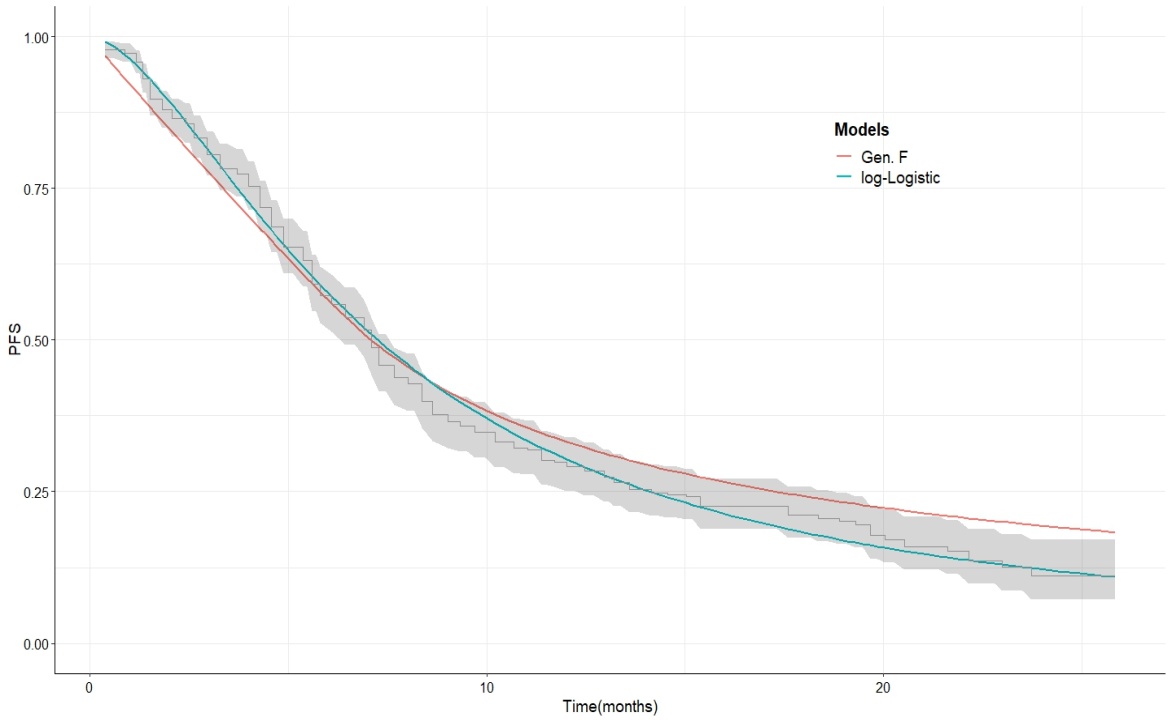


**Figure S4** Visual inspection of genf, and loglogistic distributions for PFS curve in the atezolizumab plus chemotherapy arm


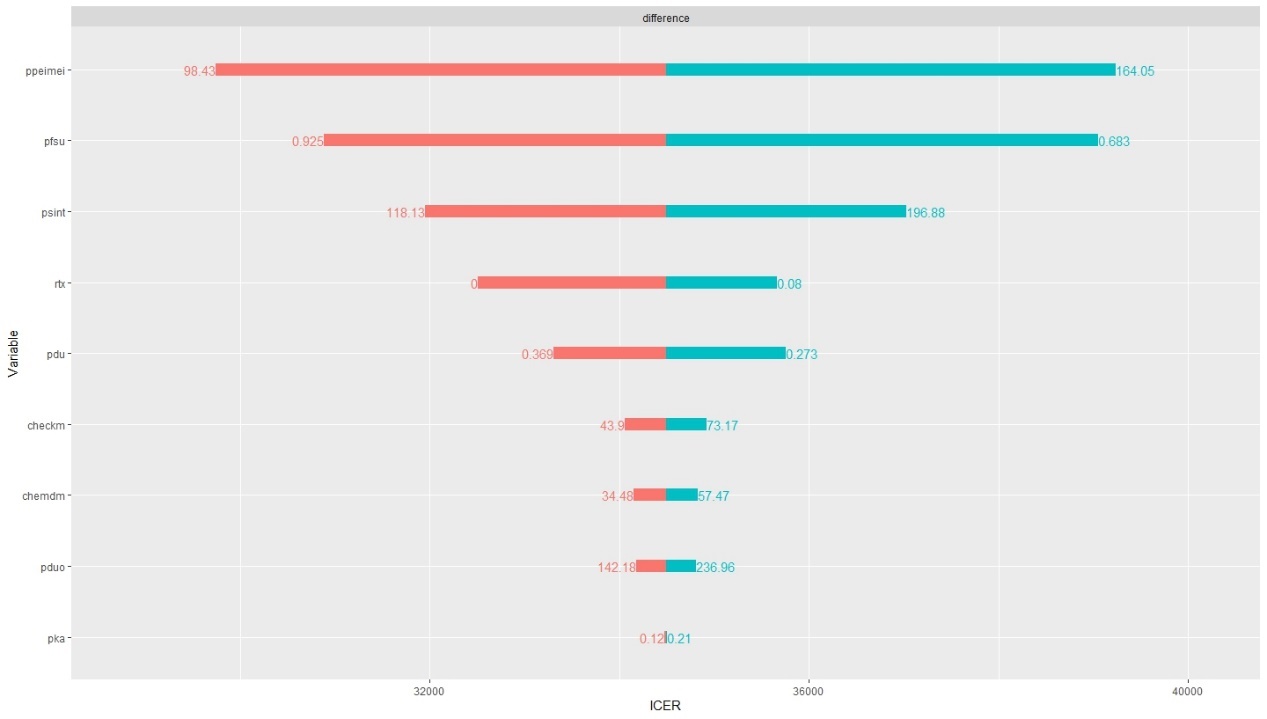


**Figure S5** Deterministic sensitivity analysis of sint-chemo versus chemo. ppeimei, price of pemetrexed; pfsu, utility of PFS; psint, price of sintilimab; rtx, discount rate; pdu, utility of PD; checkm, cost of imaging examination and laboratory test per cycle; chemdm, cost of medical service of chemotherapy per cycle; pduo, price of docetaxel; pka, price of carboplatin.


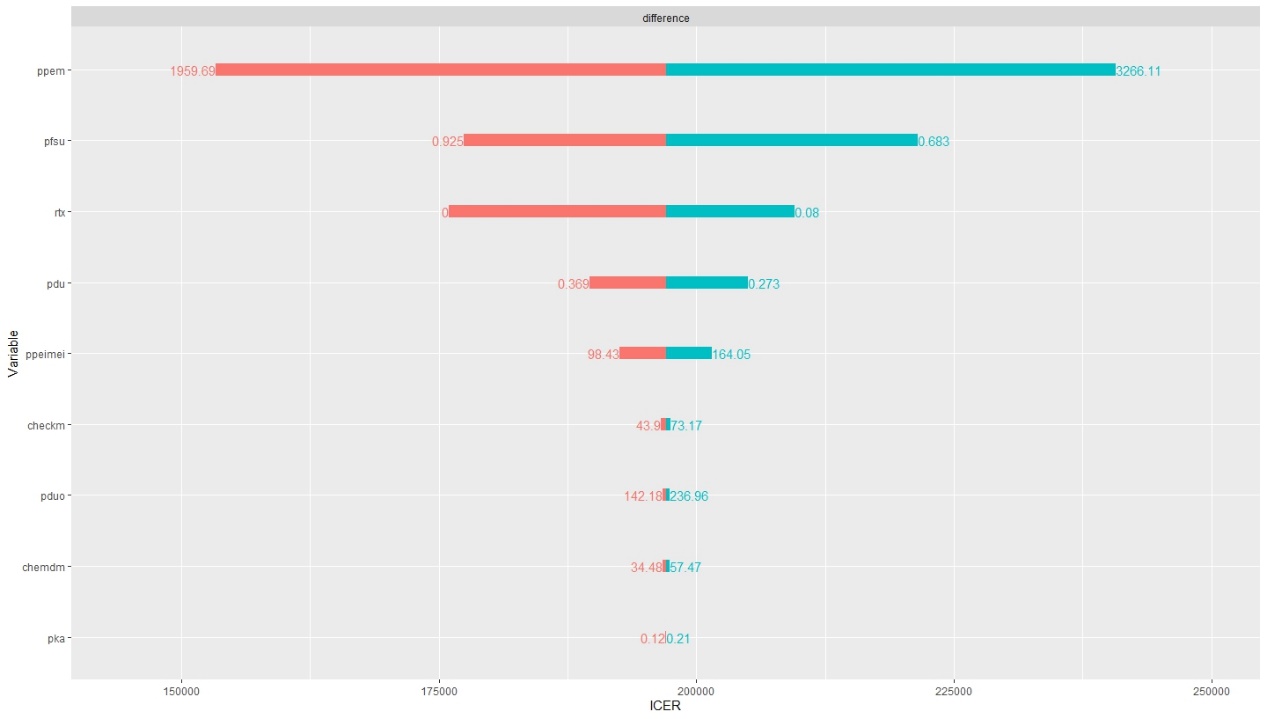


**FigureS6** Deterministic sensitivity analysis of pem-chemo versus chemo.

ppem, price of pembrolizumab; pfsu, utility of PFS; rtx, discount rate; pdu, utility of PD; ppeimei, price of pemetrexed; checkm, cost of imaging examination and laboratory test per cycle; pduo, price of docetaxel; chemdm, cost of medical service of chemotherapy per cycle; pka, price of carboplatin.


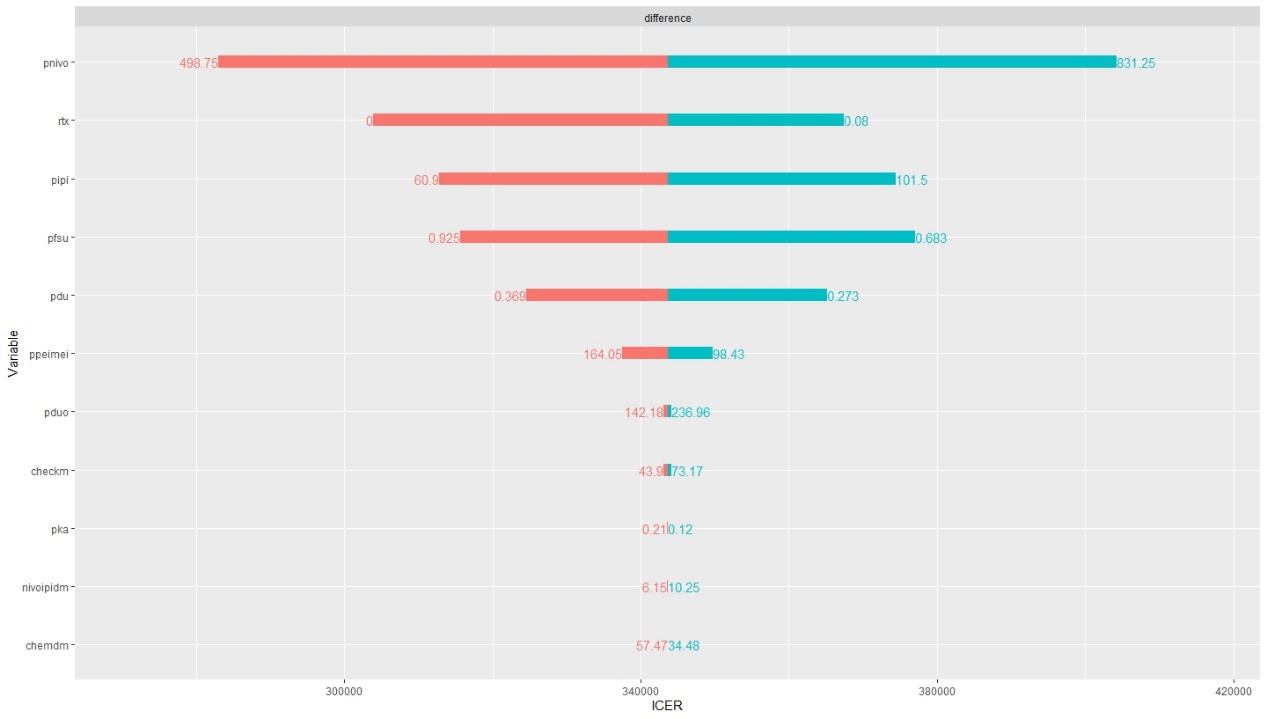


**Figure S7** Deterministic sensitivity analysis of nivo-ipi-chemo versus chemo. pnivo, price of nivolumab; rtx, discount rate; pipi, price of ipilimumab; pfsu, utility of PFS; pdu, utility of PD; ppeimei, price of pemetrexed; pduo, price of docetaxel; checkm, cost of imaging examination and laboratory test per cycle; pka, price of carboplatin; nivoipidm, cost of medical service of nivolumab plus ipilimumab therapy per cycle; chemdm, cost of medical service of chemotherapy per cycle.


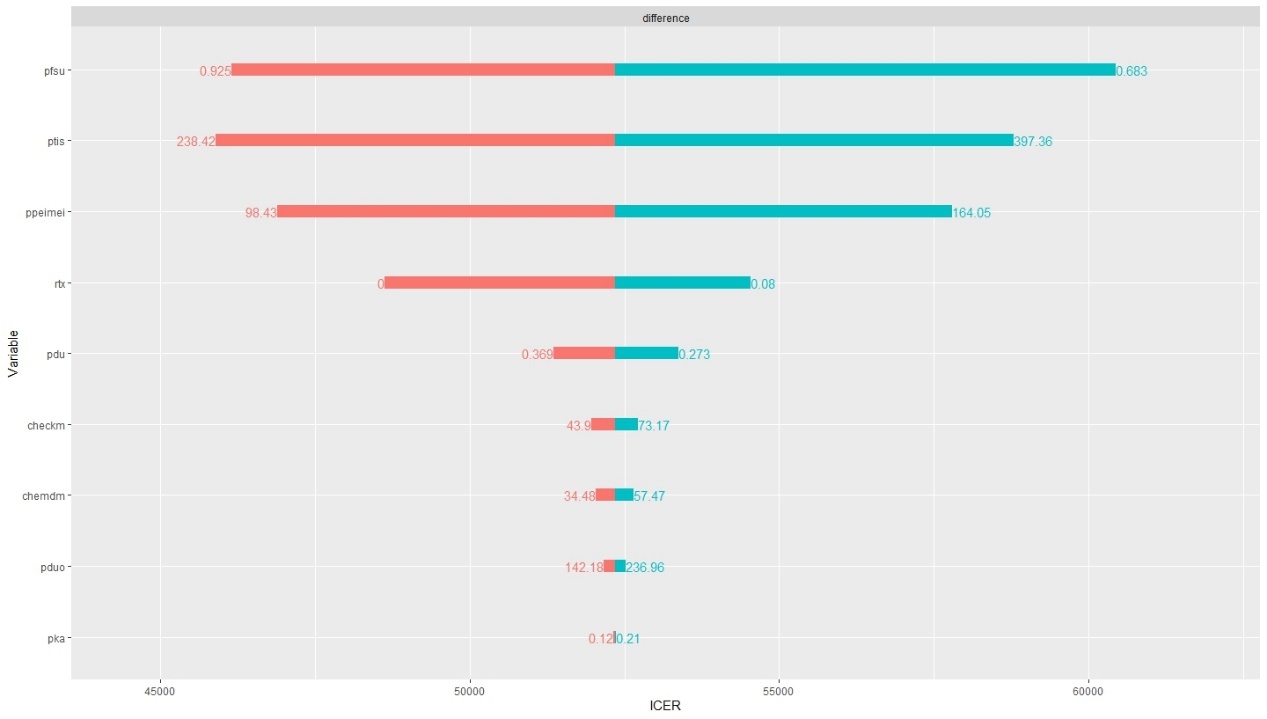


**Figure S8** Deterministic sensitivity analysis of tisle -chemo versus chemo. pfsu, utility of PFS; ptis, price of tislelizumab; ppeimei, price of pemetrexed; rtx, discount rate; pdu, utility of PD; checkm, cost of imaging examination and laboratory test per cycle; chemdm, cost of medical service of chemotherapy per cycle; pduo, price of docetaxel; pka, price of carboplatin.


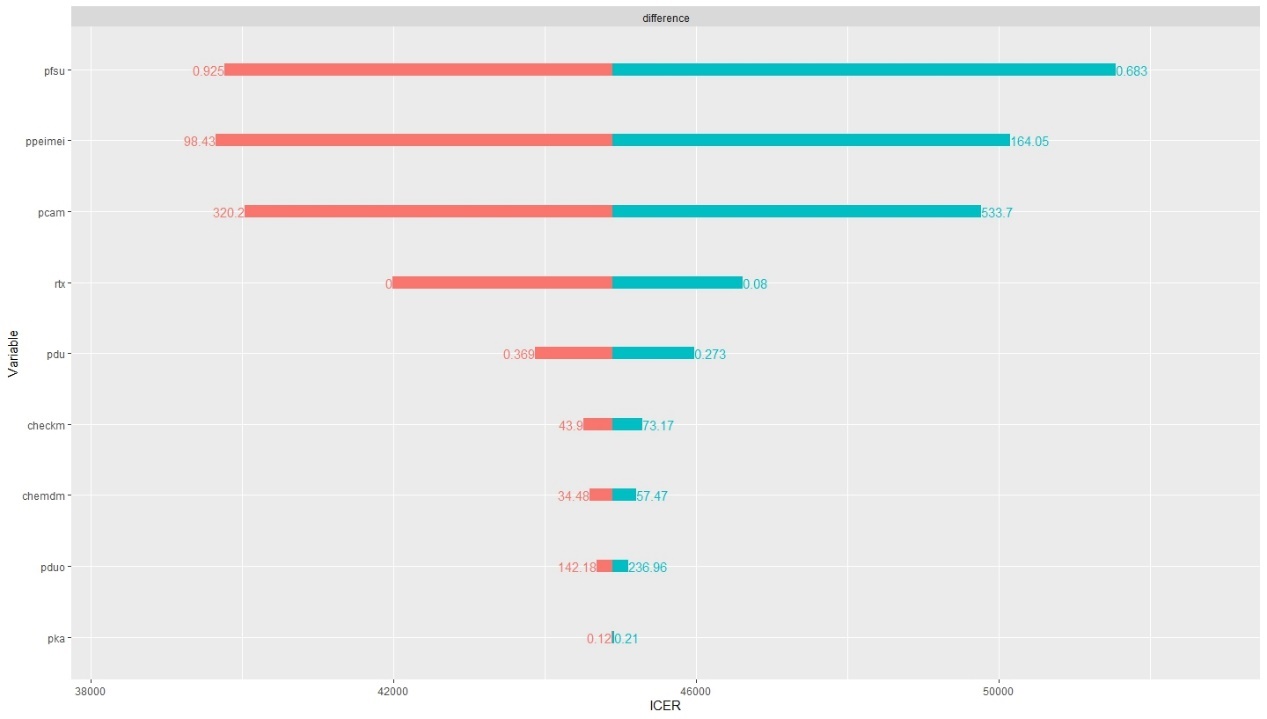


**Figure S9** Deterministic sensitivity analysis of camre-chemo versus chemo. pfsu, utility of PFS; ppeimei, price of pemetrexed; pcam, price of camrelizumab; rtx, discount rate; pdu, utility of PD; checkm, cost of imaging examination and laboratory test per cycle; chemdm, cost of medical service of chemotherapy per cycle; pduo, price of docetaxel; pka, price of carboplatin.


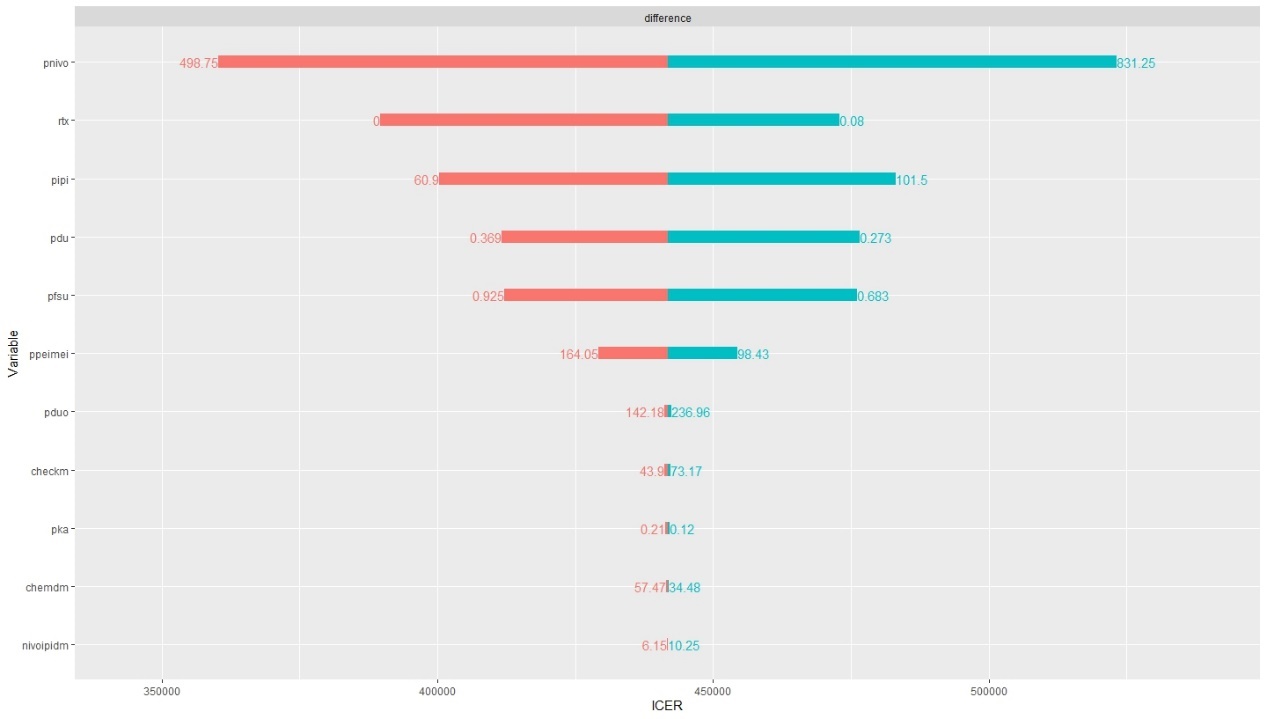


**Figure S10** Deterministic sensitivity analysis of nivo-ipi versus chemo. pnivo, price of nivolumab; rtx, discount rate; pipi, price of ipilimumab; pdu, utility of PD; pfsu, utility of PFS; ppeimei, price of pemetrexed; pduo, price of docetaxel; checkm, cost of imaging examination and laboratory test per cycle; pka, price of carboplatin; chemdm, cost of medical service of chemotherapy per cycle; nivoipidm, cost of medical service of nivolumab plus ipilimumab therapy per cycle.


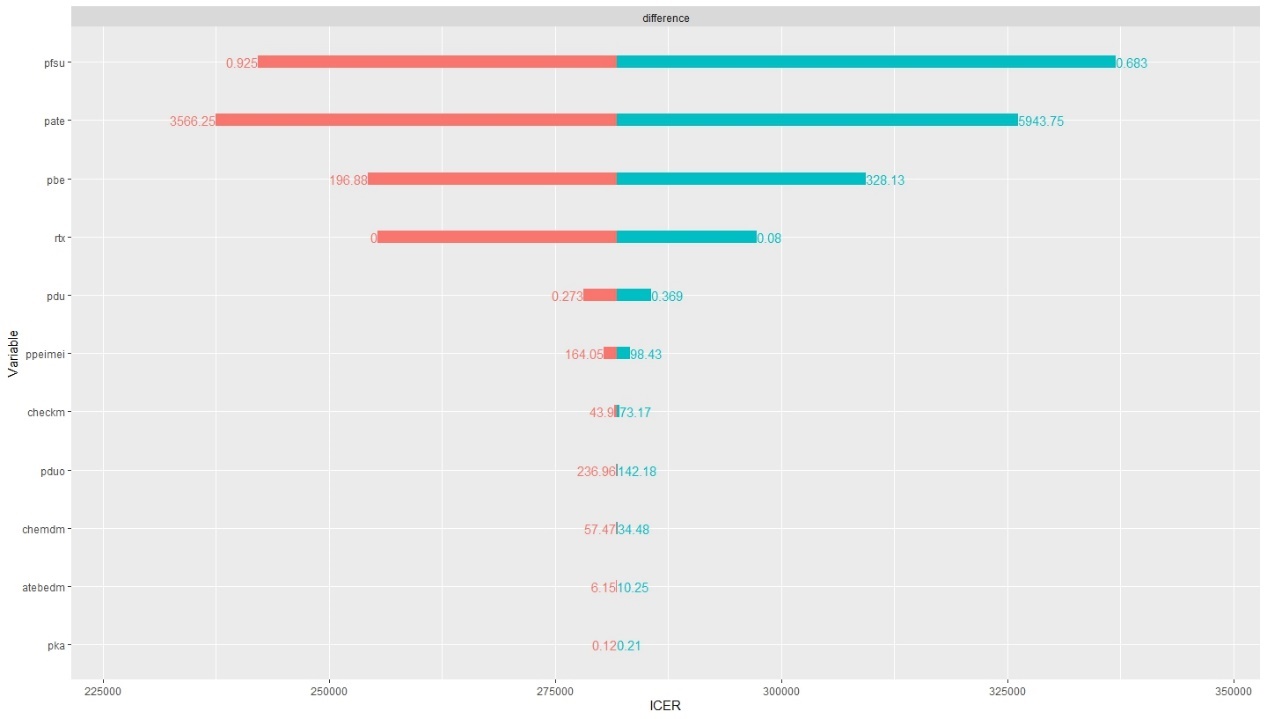


**Figure S11** Deterministic sensitivity analysis of atezo-beva-chemo versus chemo. pfsu, utility of PFS; pate, price of atezolizumab; pbe, price of bevacizumab; rtx, discount rate; pdu, utility of PD; ppeimei, price of pemetrexed; checkm, cost of imaging examination and laboratory test per cycle; pduo, price of docetaxel; chemdm, cost of medical service of chemotherapy per cycle; atebedm, cost of medical service of atezolizumab plus bevacizumab therapy per cycle; pka, price of carboplatin.


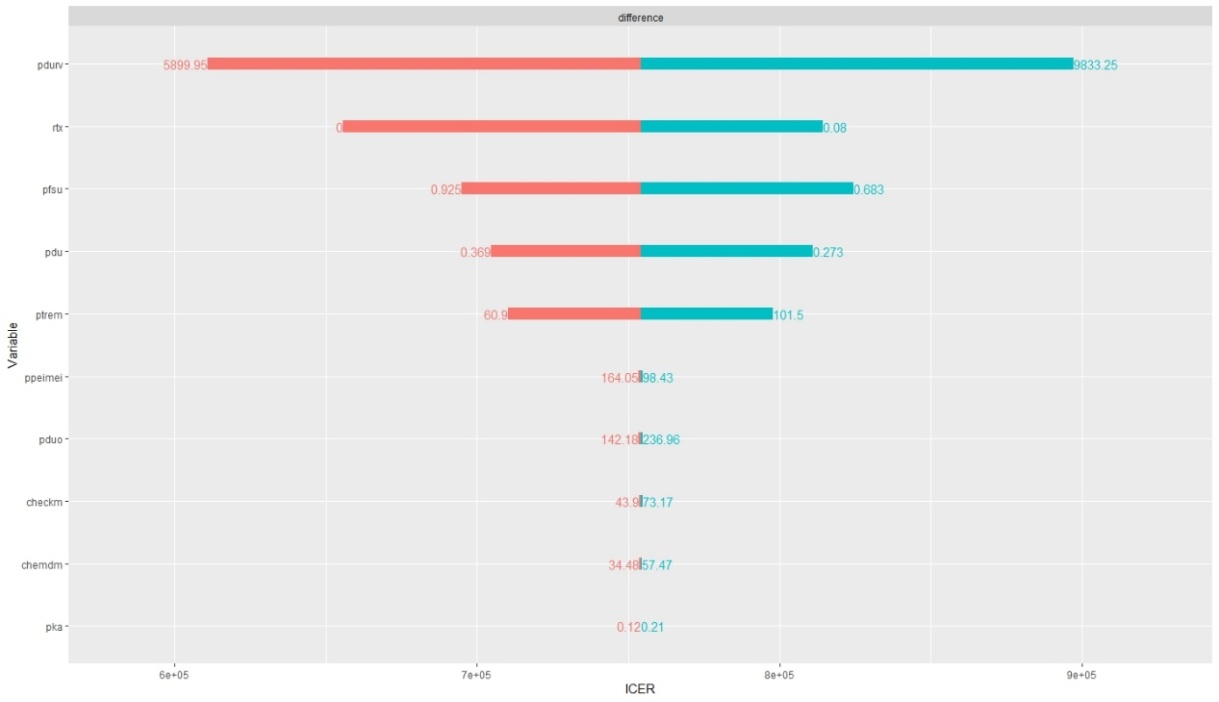


**Figure S12** Deterministic sensitivity analysis of durva-treme-chemo versus chemo. pdurv, price of durvalumab; rtx, discount rate; pfsu, utility of PFS; pdu, utility of PD; ptrem, price of tremelimumab; ppeimei, price of pemetrexed; pduo, price of docetaxel; checkm, cost of imaging examination and laboratory test per cycle; chemdm, cost of medical service of chemotherapy per cycle; pka, price of carboplatin.


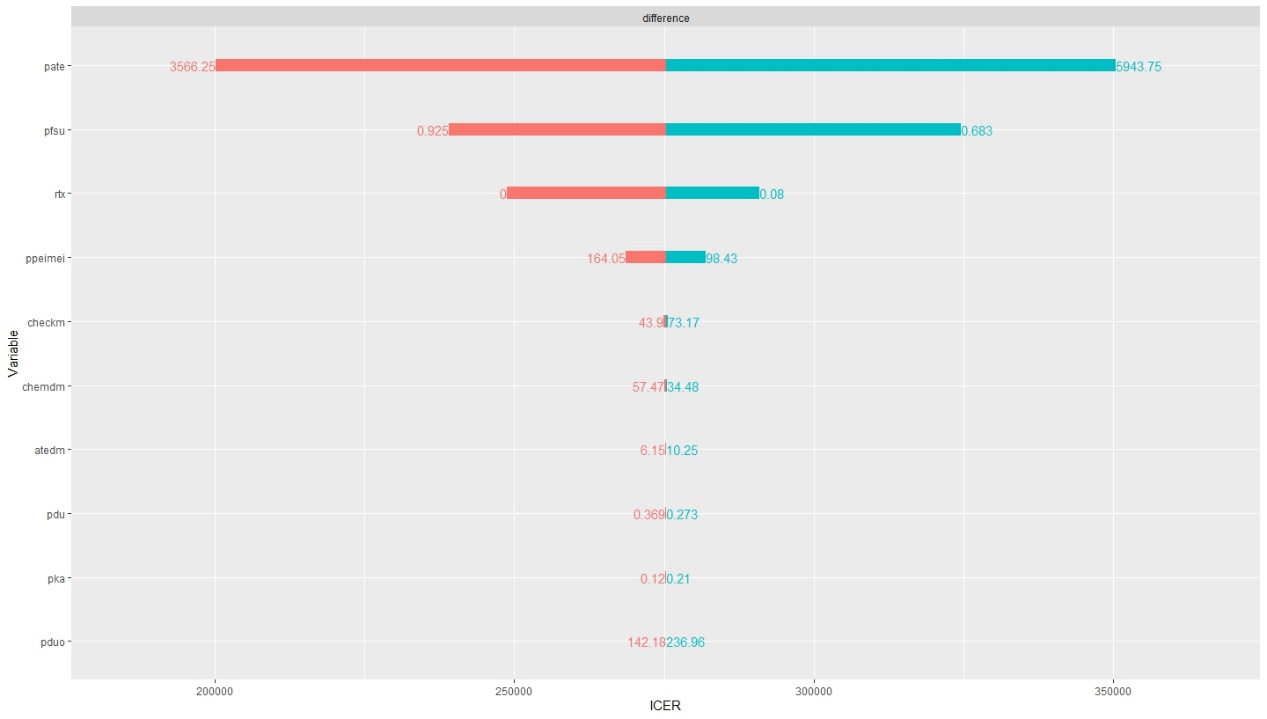


**Figure S13** Deterministic sensitivity analysis of atezo-chemo versus chemo. pate, price of atezolizumab; pfsu, utility of PFS; rtx, discount rate; ppeimei, price of pemetrexed; checkm, cost of imaging examination and laboratory test per cycle; chemdm, cost of medical service of chemotherapy per cycle; atedm, cost of medical service of atezolizumab therapy per cycle; pdu, utility of PD; pka, price of carboplatin; pduo, price of docetaxel.


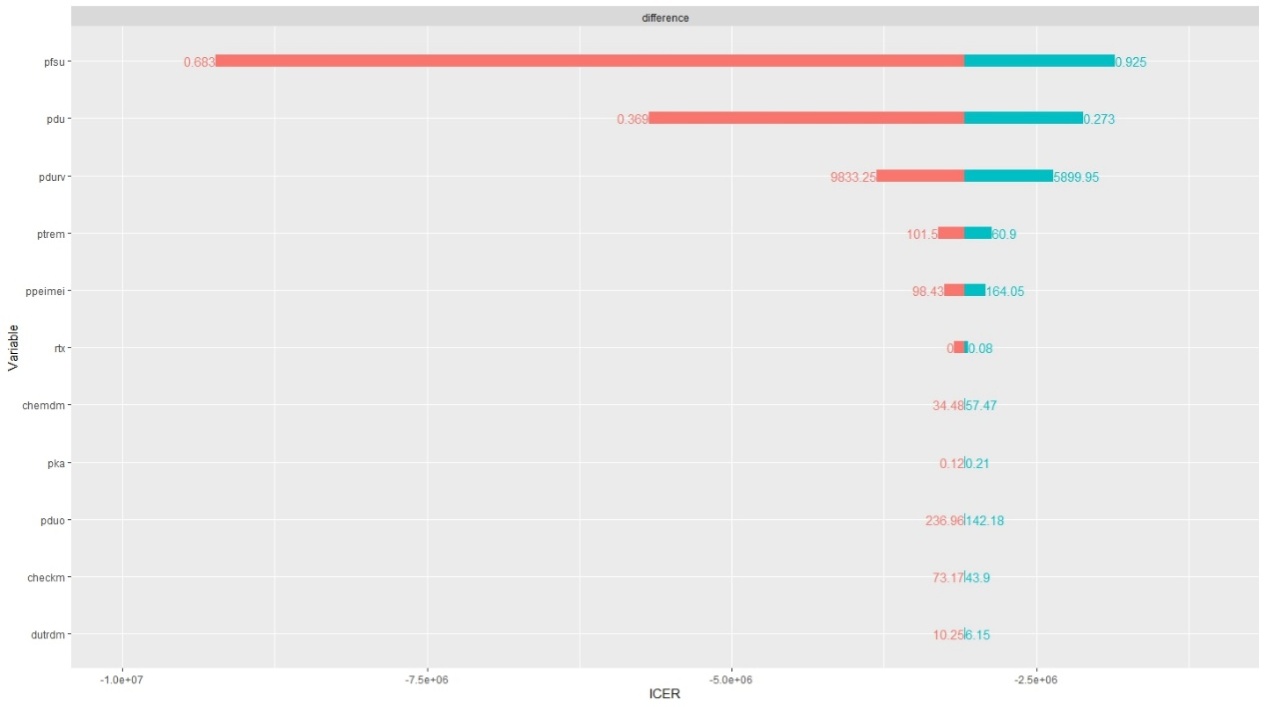


**Figure S14** Deterministic sensitivity analysis of durva-treme versus chemo. pfsu, utility of PFS; pdu, utility of PD; pdurv, price of durvalumab; ptrem, price of tremelimumab; ppeimei, price of pemetrexed; rtx, discount rate; chemdm, cost of medical service of chemotherapy per cycle; pka, price of carboplatin; pduo, price of docetaxel; checkm, cost of imaging examination and laboratory test per cycle; dutrdm, cost of medical service of durvalumab plus tremelimumab therapy per cycle.


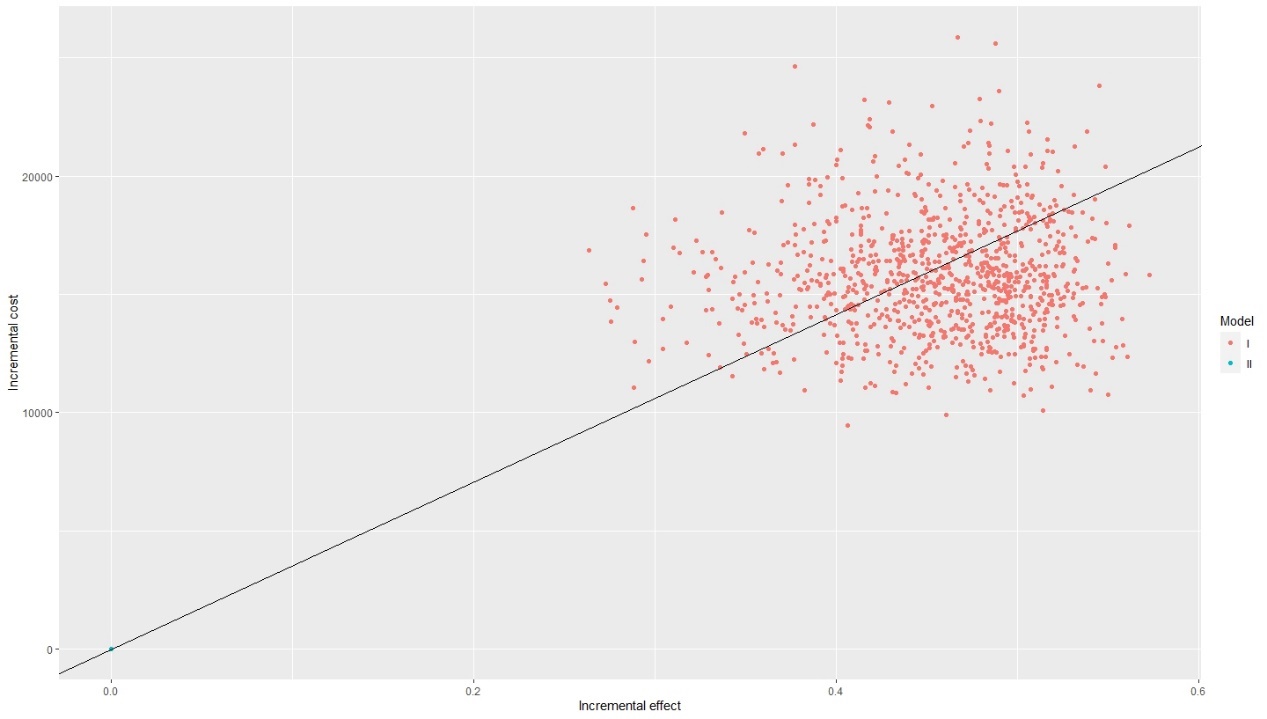


**Figure S15** Probabilistic sensitivity analysis of sint-chemo versus chemo (scatter diagram). I, sintilimab plus chemotherapy; II, chemotherapy alone; WTP=$35,424.12.


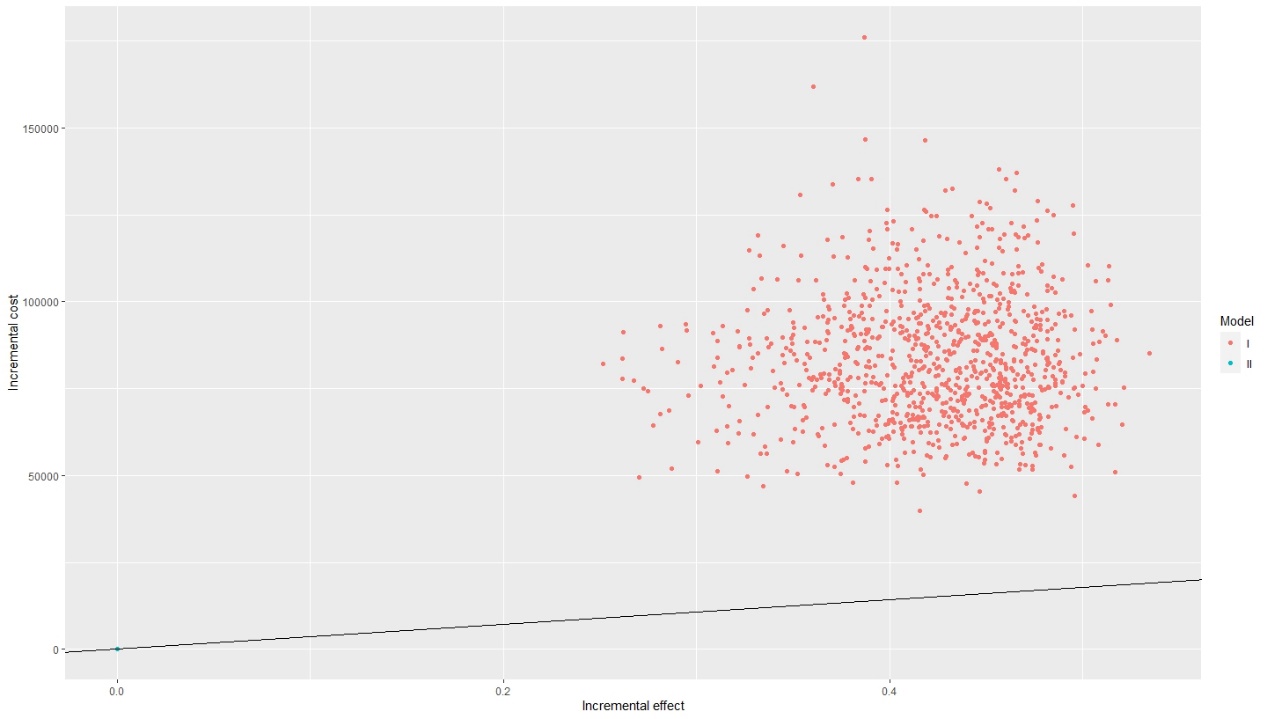


**Figure S16** Probabilistic sensitivity analysis of pem-chemo versus chemo (scatter diagram). I, pembrolizumab plus chemotherapy; II, chemotherapy alone; WTP=$35,424.12.


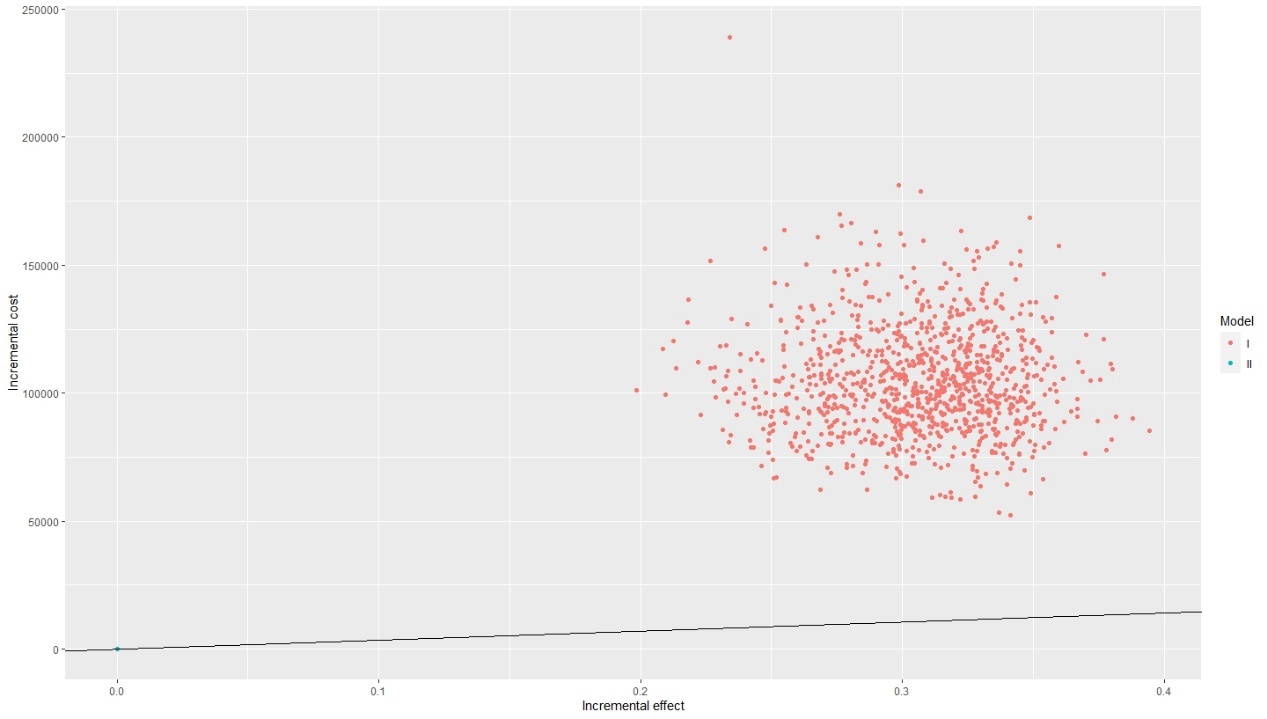


**Figure S17** Probabilistic sensitivity analysis of nivo-ipi-chemo versus chemo (scatter diagram). I, nivolumab plus ipilimumab plus chemotherapy; II, chemotherapy alone; WTP=$35,424.12.


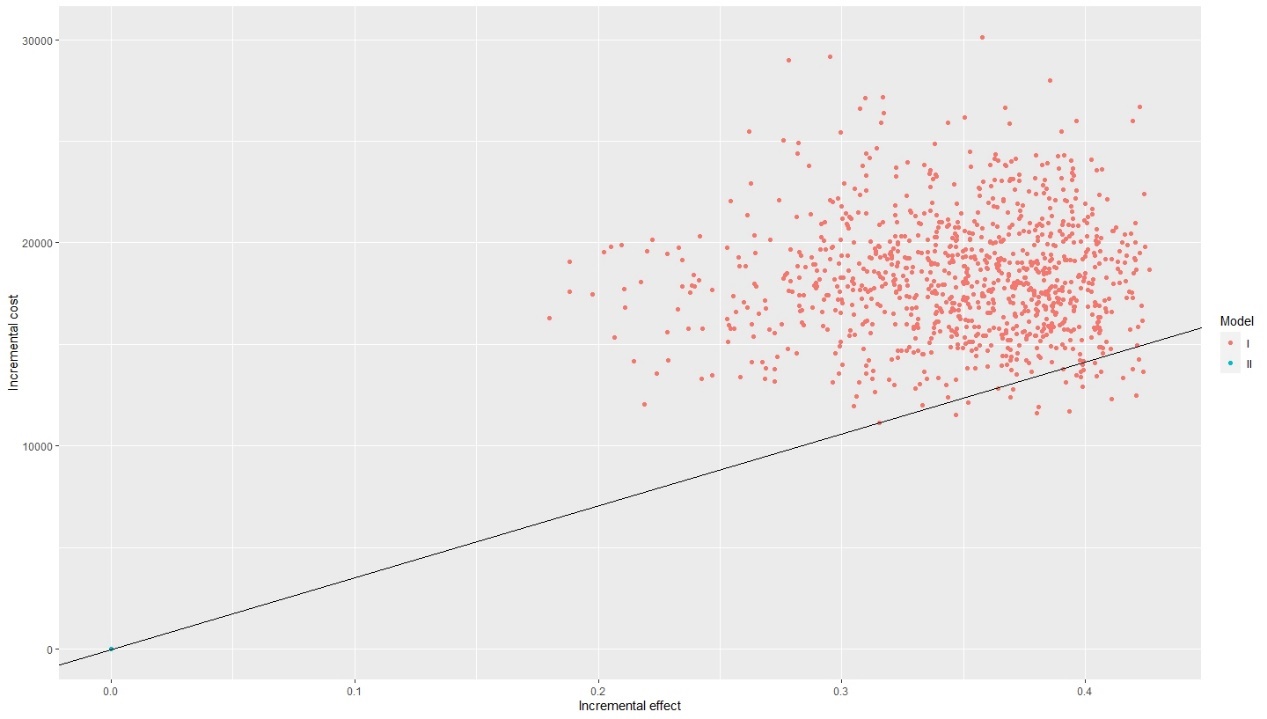


**Figure S18** Probabilistic sensitivity analysis of tisle-chemo versus chemo (scatter diagram). I, tislelizumab plus chemotherapy; II, chemotherapy alone; WTP=$35,424.12.


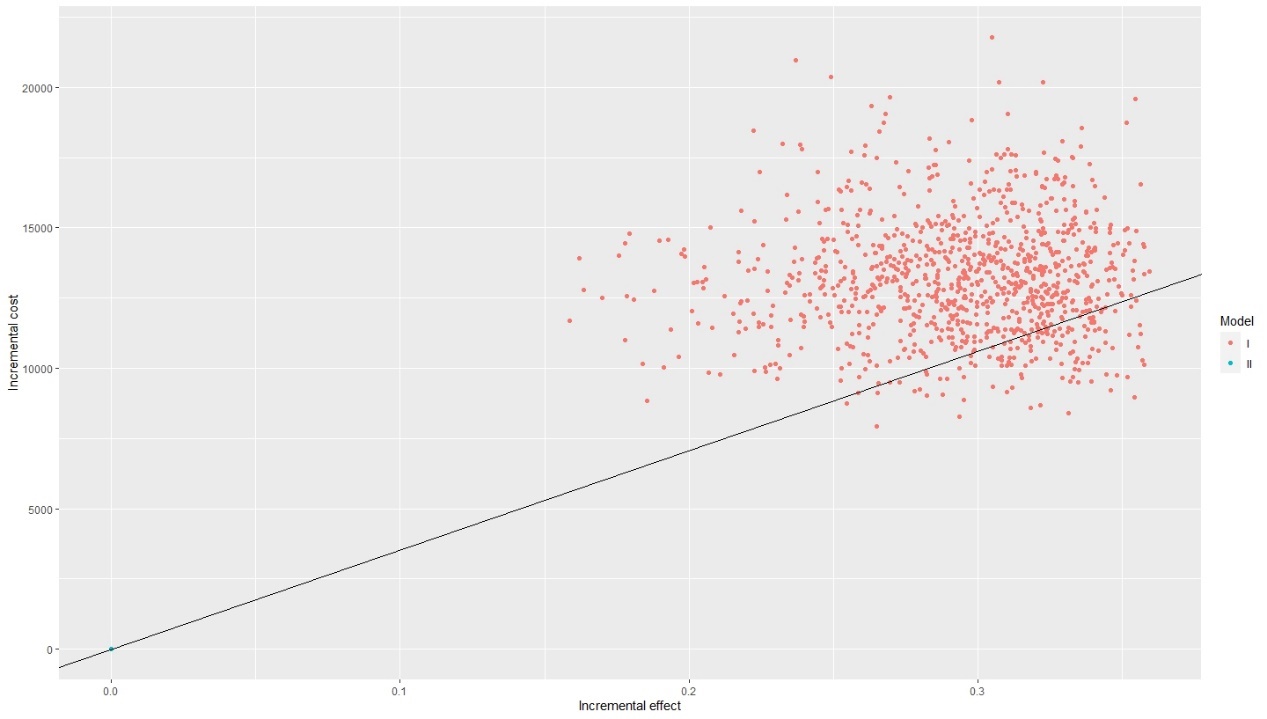


**Figure S19** Probabilistic sensitivity analysis of camre-chemo versus chemo (scatter diagram). I, camrelizumab plus chemotherapy; II, chemotherapy alone; WTP=$35,424.12.


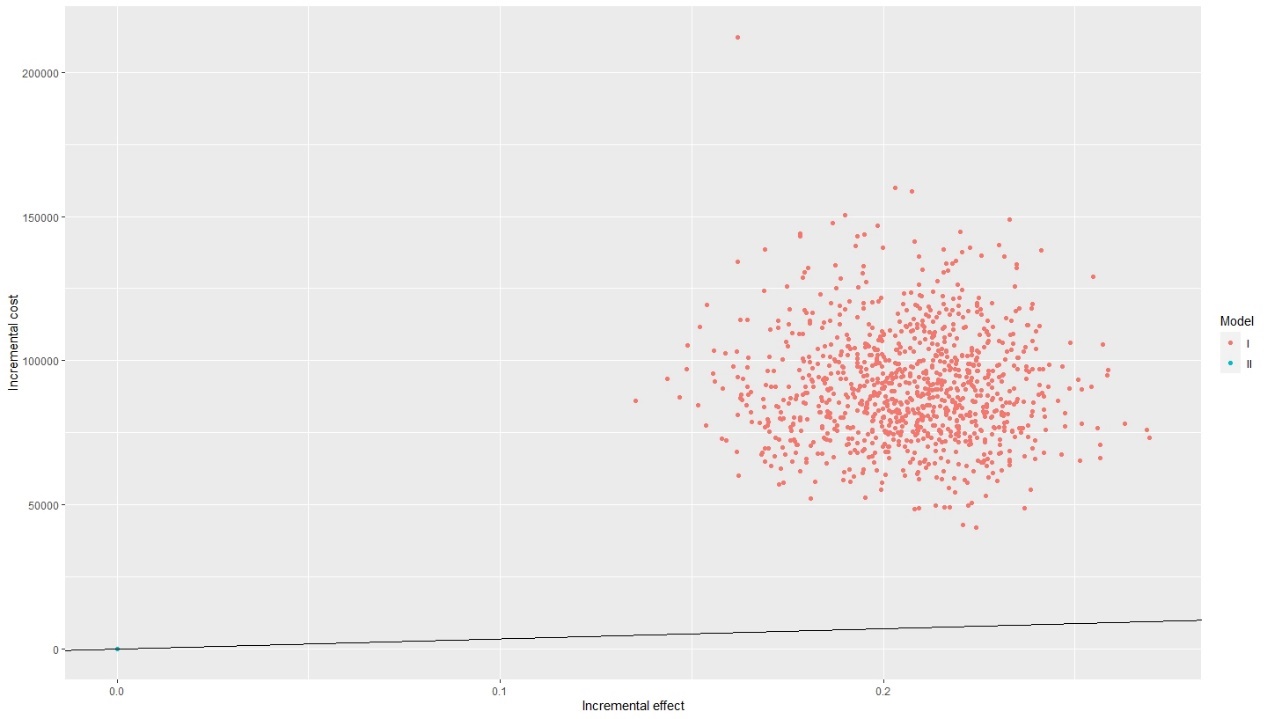


**Figure S20** Probabilistic sensitivity analysis of nivo-ipi versus chemo (scatter diagram). I, nivolumab plus ipilimumab; II, chemotherapy alone; WTP=$35,424.12.


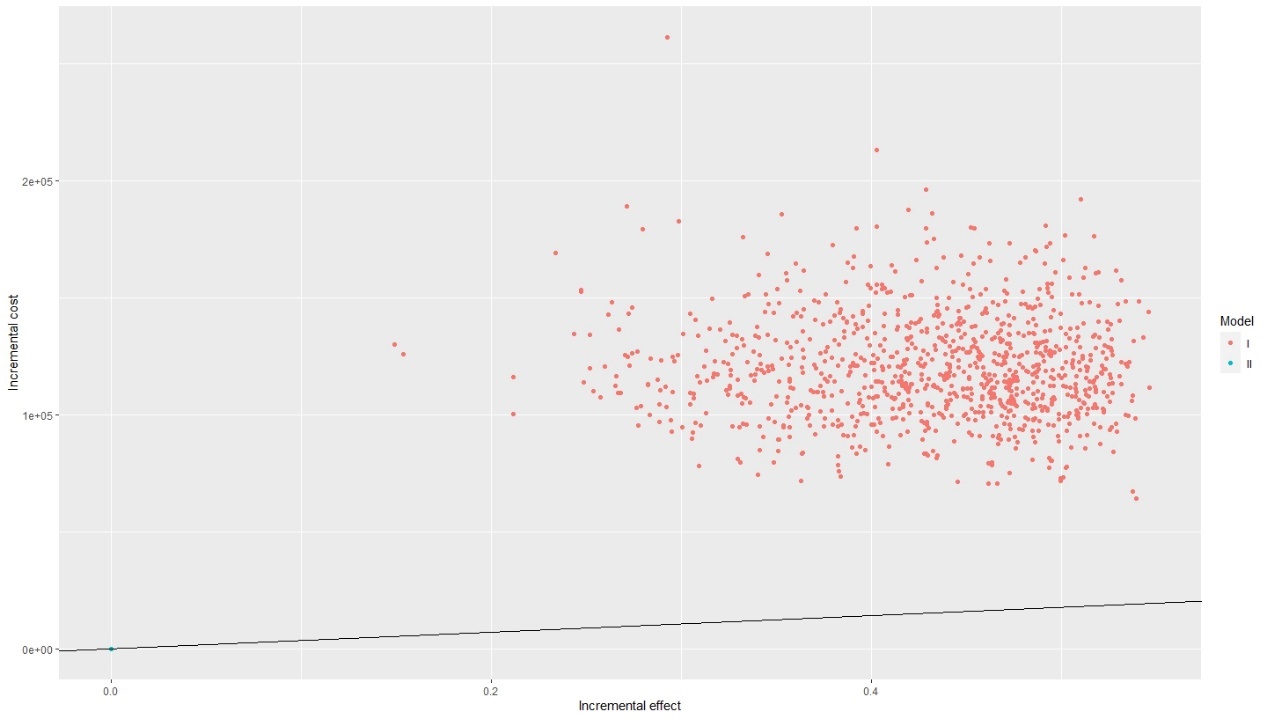


**Figure S21** Probabilistic sensitivity analysis of atezo-beva-chemo versus chemo (scatter diagram). I, atezolizumab plus bevacizumab plus chemotherapy; II, chemotherapy alone; WTP=$35,424.12.


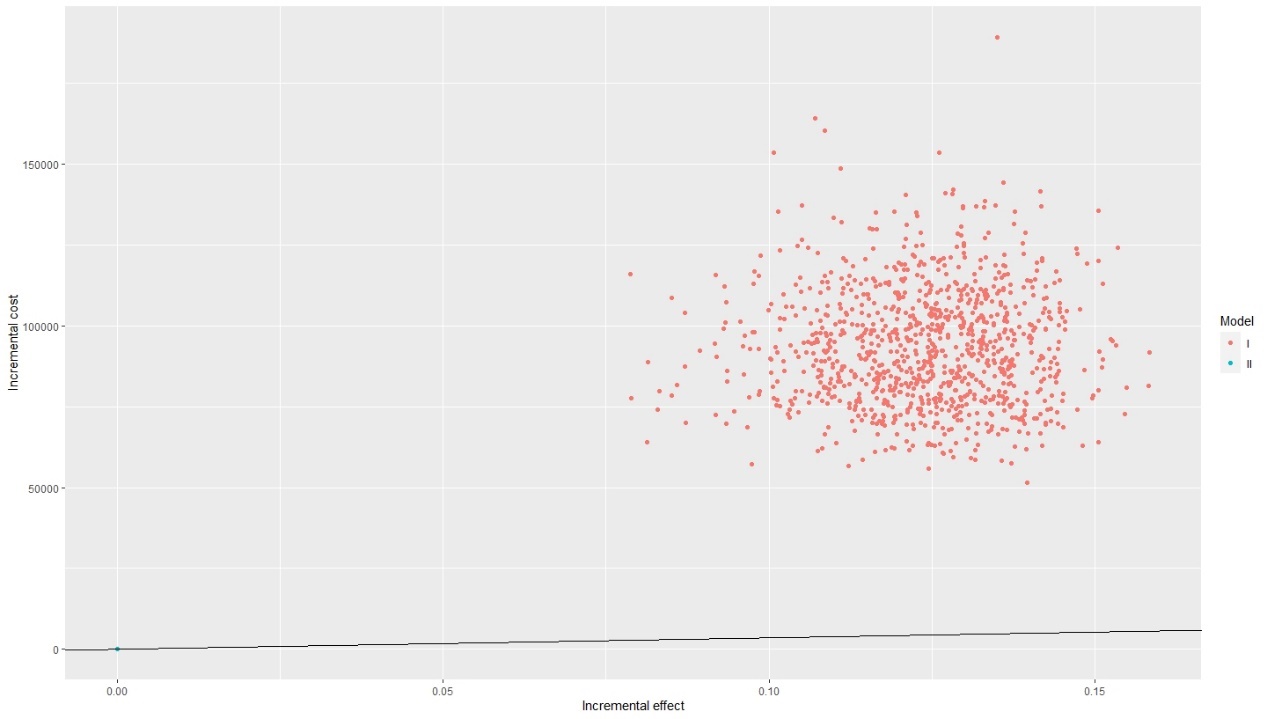


**Figure S22** Probabilistic sensitivity analysis of durva-treme-chemo versus chemo (scatter diagram). I, durvalumab plus tremelimumab plus chemotherapy; II, chemotherapy alone; WTP=$35,424.12.


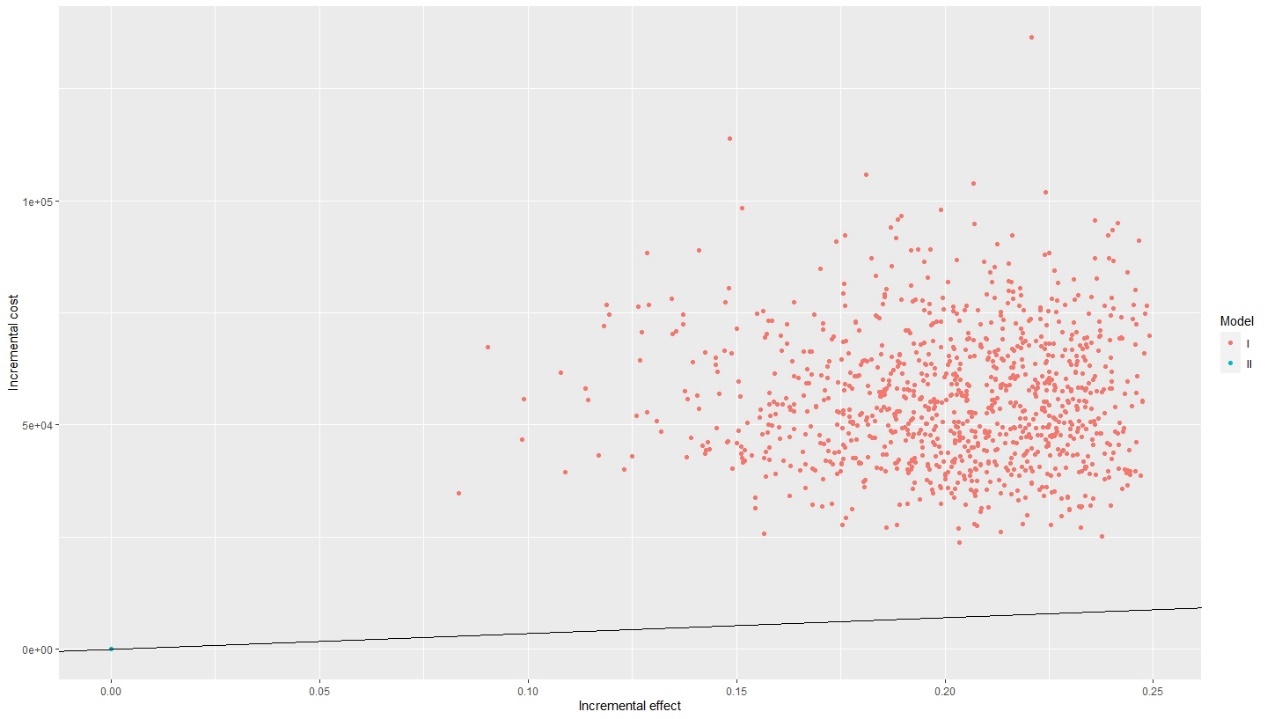


**Figure S23** Probabilistic sensitivity analysis of atezo-chemo versus chemo (scatter diagram). I, atezolizumab plus chemotherapy; II, chemotherapy alone; WTP=$35,424.12.


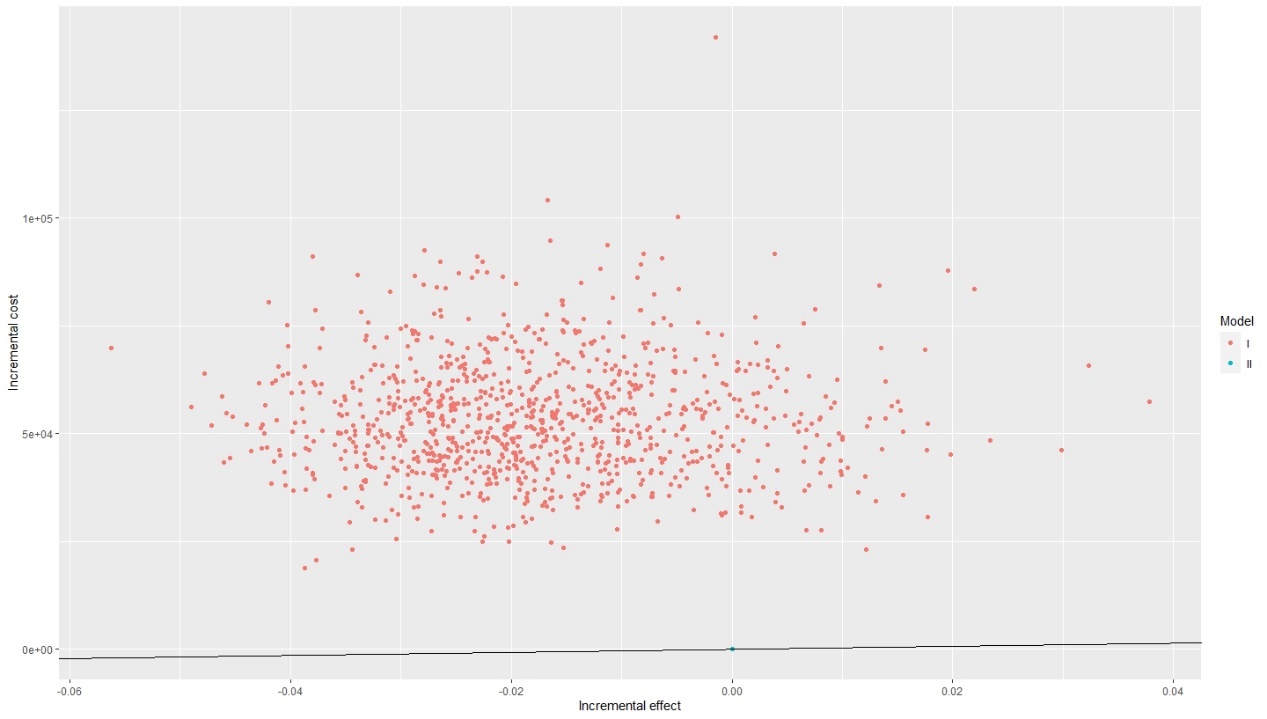


**Figure S24** Probabilistic sensitivity analysis of durva-treme versus chemo (scatter diagram). I, durvalumab plus tremelimumab; II, chemotherapy alone; WTP=$35,424.12.

**Abbreviations**

SAE: serious adverse event; sint-chemo: sintilimab plus chemotherapy; pem-chemo: pembrolizumab plus chemotherapy; nivo-ipi-chemo: nivolumab plus ipilimumab plus chemotherapy; tisle-chemo: tislelizumab plus chemotherapy; camre-chemo: camrelizumab plus chemotherapy; atezo-beva-chemo: atezolizumab plus bevacizumab plus chemotherapy; durva-treme-chemo: durvalumab plus tremelimumab plus chemotherapy; ate-chemo: atezolizumab plus chemotherapy; durva-treme: durvalumab plus tremelimumab; chemo: chemotherapy.

**References**

1. Guan HJ, Liu G, Xie F, Sheng YN, Shi LW. Cost-Effectiveness of Osimertinib as a Second-Line Treatment in Patients With EGFR-Mutated Advanced Non-Small Cell Lung Cancer in China. Clin Ther. 2019; 41(11):2308-2320.e11.

2. Shi X, Zhu QY. Cost-Utility Analysis of Afatinib and Gefitinib in First-Line Treatment of EGFR Mutation-Positive Non-Small Cell Lung Cancer. Chin J Mod Appl Pharm. 2019; 36(21):2701-6.

3. Xu H, Ma AX. Cost-Effectiveness Analysis of Pembrolizumab Versus Chemotherapy as First-Line Treatment in Non-Small Cell Lung Cancer With Different PD-L1 Expression Levels Based on Partitioned Survival Model. Chin J Hosp Pharm. 2020; 40:1-8.

4. Shi FH, Meng R, Wang ZJ, Rui MJ, Shang Y, Ma AX. Cost-effectiveness analysis of applying camrelizumab as second-line therapy for the treatment of advanced hepatocellular carcinoma. Chin Heal Econ. 2021; 40(2): 62-65.

5. Hou YL, Wu B. Atezolizumab plus bevacizumab versus sorafenib as first-line treatment for unresectable hepatocellular carcinoma: a cost-effectiveness analysis. Cancer Commun (Lond). 2020; 40(12):743-745.

6. Kang S, Wang XC, Zhang Y, Zhang BY, Shang FJ, Guo W. First-Line Treatments for Extensive-Stage Small-Cell Lung Cancer With Immune Checkpoint Inhibitors Plus Chemotherapy: A Network Meta-Analysis and Cost-Effectiveness Analysis. Front Oncol. 2022;11:1-11.

7. Guan HJ, Wang CP, Chen C, Han S, Zhao ZG. Cost-Effectiveness of 12 First-Line Treatments for Patients With Advanced EGFR Mutated NSCLC in the United Kingdom and China. Front Oncol. 2022;12:1-12.

8. Nafees B, Lloyd AJ, Dewilde S, Rajan N, Lorenzo M. Health State Utilities in Non-Small Cell Lung Cancer: An International Study. Asia Pac J Clin Oncol. 2017; 13:e195- e203.

9. Zeng XH, Wan XM, Peng LB, Peng Y, Ma F, Liu Q, et al. Cost-effectiveness analysis of pembrolizumab plus chemotherapy for previously untreated metastatic non-small cell lung cancer in the USA. BMJ Open. 2019;9(12): 1-6.
